# Risk-associated alterations in marrow T cells in pediatric leukemia

**DOI:** 10.1172/jci.insight.140179

**Published:** 2020-08-20

**Authors:** Jithendra Kini Bailur, Samuel S. McCachren, Katherine Pendleton, Juan C. Vasquez, Hong Seo Lim, Alyssa Duffy, Deon B. Doxie, Akhilesh Kaushal, Connor Foster, Deborah DeRyckere, Sharon Castellino, Melissa L. Kemp, Peng Qiu, Madhav V. Dhodapkar, Kavita M. Dhodapkar

**Affiliations:** 1 Department of Hematology/Oncology, Emory University School of Medicine, Emory University, Atlanta, Georgia, USA.; 2 The Wallace H. Coulter Department of Biomedical Engineering, Georgia Institute of Technology and Emory University, Atlanta, Georgia, USA.; 3 Aflac Cancer and Blood Disorders Center, Children’s Healthcare of Atlanta, Emory University, Atlanta, Georgia, USA.; 4 Yale University School of Medicine, Yale University, New Haven, Connecticut, USA.; 5 Winship Cancer Institute, Atlanta, Georgia, USA.

**Keywords:** Immunology, Leukemias, T cells

## Abstract

Current management of childhood leukemia is tailored based on disease risk determined by clinical features at presentation. Whether properties of the host immune response impact disease risk and outcome is not known. Here, we combine mass cytometry, single cell genomics, and functional studies to characterize the BM immune environment in children with B cell acute lymphoblastic leukemia and acute myelogenous leukemia at presentation. T cells in leukemia marrow demonstrate evidence of chronic immune activation and exhaustion/dysfunction, with attrition of naive T cells and TCF1^+^ stem-like memory T cells and accumulation of terminally differentiated effector T cells. Marrow-infiltrating NK cells also exhibit evidence of dysfunction, particularly in myeloid leukemia. Properties of immune cells identified distinct immune phenotype–based clusters correlating with disease risk in acute lymphoblastic leukemia. High-risk immune signatures were associated with expression of stem-like genes on tumor cells. These data provide a comprehensive assessment of the immune landscape of childhood leukemias and identify targets potentially amenable to therapeutic intervention. These studies also suggest that properties of the host response with depletion of naive T cells and accumulation of terminal-effector T cells may contribute to the biologic basis of disease risk. Properties of immune microenvironment identified here may also impact optimal application of immune therapies, including T cell–redirection approaches in childhood leukemia.

## Abstract 2

In high risk pediatric leukemia, naive and stem-like memory T cells are depleted and terminally differentiated effector T cells accumulate.

## Graphical abstract

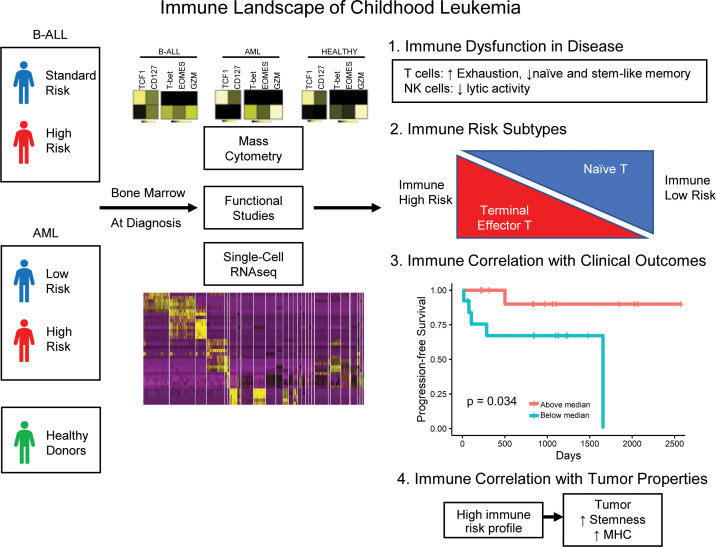

## Introduction

Acute leukemias are a leading cause of death from cancer before age 20 in the United States (1). Both B cell acute lymphoblastic leukemia (B-ALL) and acute myeloid leukemia (AML) comprise multiple entities with a distinct constellation of somatic genetic alterations, including aneuploidy, chromosome translocations, and somatic mutations (2–4). While the outcome for children with leukemia has improved considerably in the past 4 decades, current chemotherapies may carry significant risk of long-term toxicities; therefore, newer approaches using less toxic regimens are urgently needed (5).

Treatments harnessing the immune system have emerged as attractive new approaches to treat cancer and carry lower risk of long-term toxicities (6). Immune-based approaches such as redirection of T cells to tumors with chimeric antigen receptors (CARs) have led to impressive and durable responses in children with B-ALL (7). It is increasingly appreciated, mostly from studies in adult cancer, that properties of the immune microenvironment are a critical determinant of outcome following immune therapies (8, 9). In contrast to adult cancers, pediatric tumors express fewer somatic mutations and, consequently, fewer neoantigens (10–12). While low neoantigen load was initially predicted to underlie “low immunogenicity” of pediatric tumors (10–12), the presence of neoantigen-specific T cells in childhood leukemia has been described, consistent with immune recognition of these tumors (13). Comprehensive analyses of the immune microenvironment of the BM in childhood leukemias are limited, with most studies focused on a limited set of markers (13–17). Understanding the immunobiology of the tumor microenvironment will be essential for the development of effective immune therapies in childhood leukemia.

Current management of leukemias is guided by their “risk status” (1). Genomic features of leukemic cells alone are insufficient predictors of disease risk, and current risk classification in B-ALL uses, in addition, clinical features such as age and WBC count at presentation and response to therapy. Risk status in AML is also affected by genetic features of tumor cells, as well as response to therapy. Whether properties of the host immune response impact disease risk or outcome in childhood leukemia is not known.

In this study, we have combined single cell mass cytometry, single cell transcriptomics, and functional studies to characterize BM immune cells infiltrating pediatric leukemias. Our studies identify several distinct changes in the immune microenvironment of these tumors that may be amenable to therapeutic intervention by harnessing tumor immunity. We also uncover distinct immune subtypes of childhood leukemia, suggesting that immune response may contribute to the biologic basis of disease risk.

## Results

In order to characterize the BM immune microenvironment of pediatric leukemia, we analyzed BM mononuclear cells (BMMNCs) from 64 patients with newly diagnosed acute leukemia (B-ALL; *n* = 36, AML; *n* = 28), and 11 healthy donors (HD) (clinical characteristics in Supplemental Table 1; supplemental material available online with this article; https://doi.org/10.1172/jci.insight.140179DS1). Changes in immune cells were analyzed by high-dimensional mass cytometry. The mass cytometry findings were further validated using single cell RNA sequencing (scRNA-Seq) and functional studies.

### Changes in T cells.

**Figure 1:**
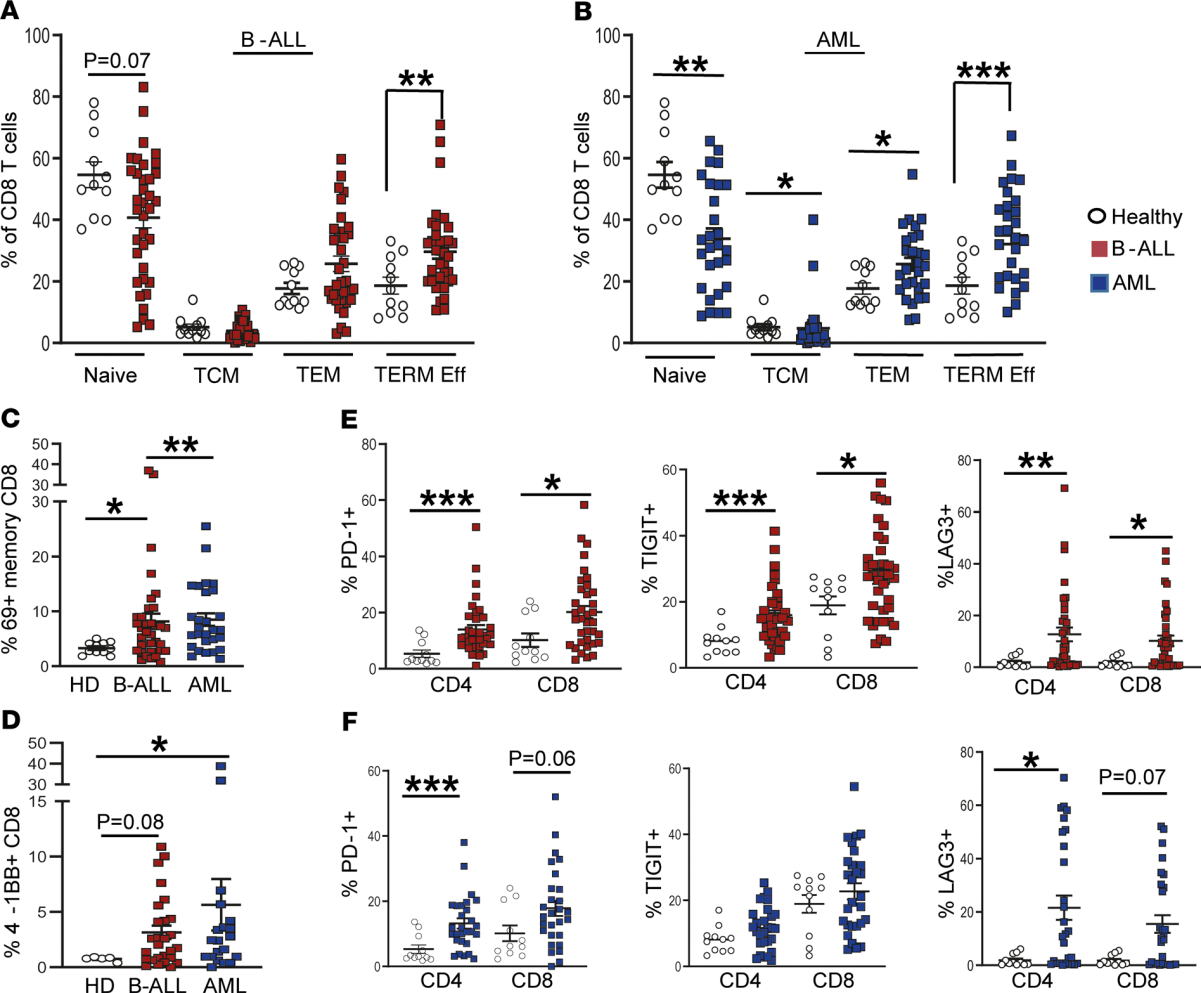
Differences in BM T cells in children with B-ALL and AML at diagnosis. BM mononuclear cells (BMMNCs) from patients with B-ALL (*n* = 36), AML (*n* = 28), and healthy donors (*n* = 11; *n* = 5 for 4-1BB) were characterized using single cell mass cytometry. (**A**) Figure shows percent naive (CCR7^+^CD45RO^–^), central memory (TCM; CCR7^+^CD45RO^+^), effector memory (TEM; CCR7^–^CD45RO^+^), and terminal effector (TERM Eff; CCR7^–^CD45RO^–^) CD8^+^ T cells in B-ALL and HD BM. (**B**) Percent naive, central memory, effector memory, and terminal effector CD8^+^ T cells in AML and HD BM. (**C**) Expression of CD69 on memory CD8^+^ T cells from B-ALL, AML, and HD marrow. (**D**) CD8^+^ T cells expressing 4-1BB in B-ALL, AML, and HD BM. (**E**) Figure shows expression of inhibitory immune checkpoints PD-1, TIGIT, and LAG3 on CD4^+^ and CD8^+^ T cells in B-ALL and HD BMMNCs. (**F**) Expression of inhibitory immune checkpoints PD-1, TIGIT, and LAG3 in CD4^+^ and CD8^+^ T cells from AML and HD BM. All graphs show mean ± SEM. *P < 0.05, **P < 0.01, ***P < 0.001 by Mann-Whitney *U* test.

CD3^+^ T cells as a proportion of total BMMNCs were lower in the leukemic marrow relative to HD, as expected, due to leukemic cell infiltration (Supplemental Figure 1A). Within the CD3^+^ T cell compartment, the proportion of CD4^+^ and CD8^+^ subsets was comparable between HD and patients with B-ALL or AML (Supplemental Figure 1B). However, in both leukemic cohorts, there was a decline in the proportion of CD8^+^ but not CD4^+^ naive T cells and an increase in terminal effector CD8^+^ T cells (Figure 1, A and B, and Supplemental Figure 1, C and D). This was associated with increased activation of memory CD8^+^ T cells, with upregulation of activation marker CD69 (Figure 1C). Together, these data indicate that T cells in the leukemic BM exhibit evidence of T cell activation and increased effector differentiation in situ, particularly within the CD8^+^ compartment, along with relative decline in naive T cells.

**Figure 2:**
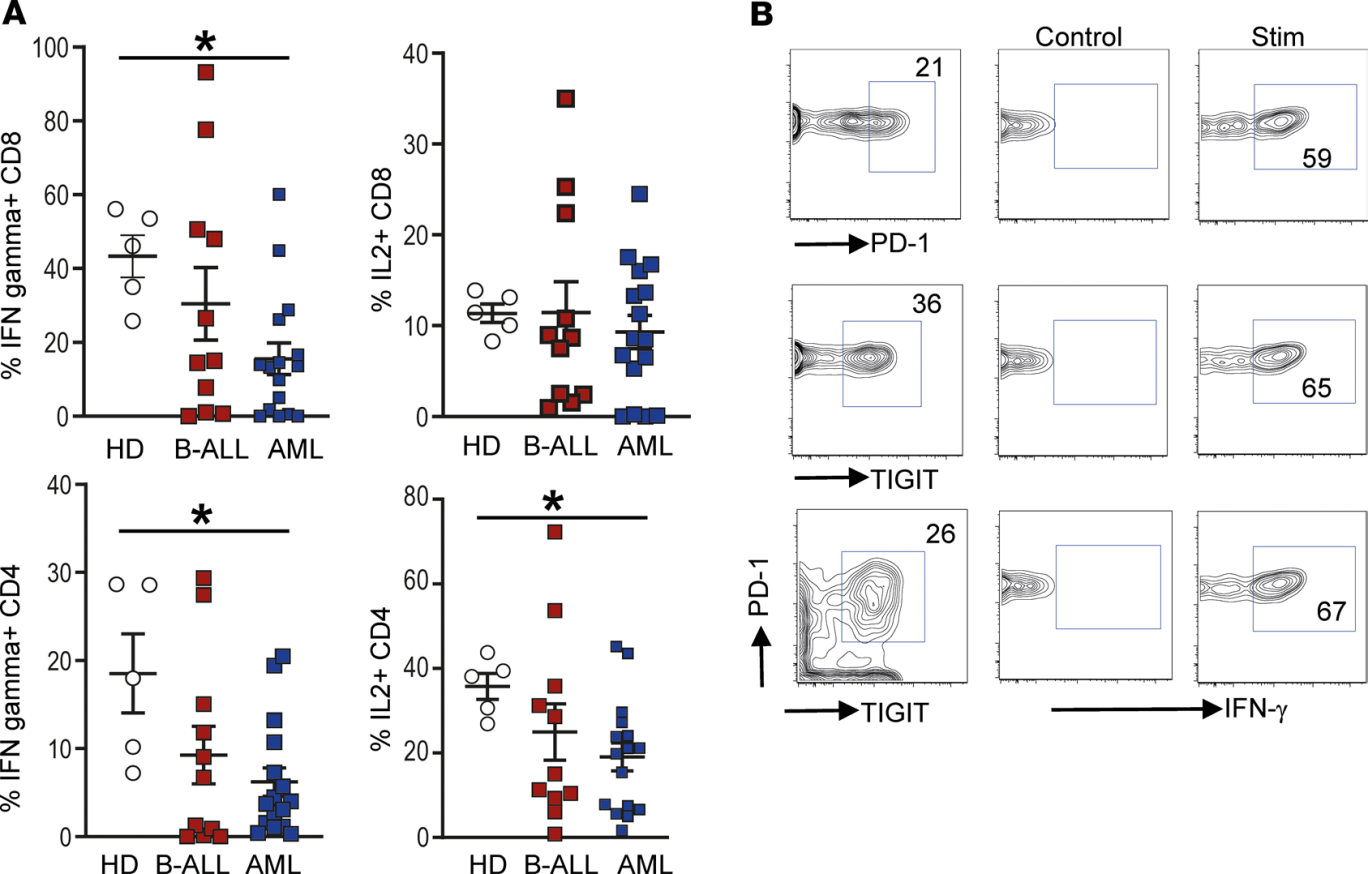
Changes in BM T cell function in children with B-ALL and AML at diagnosis. BMMNCs from B-ALL (*n* = 13), AML (*n* = 17), or HD (*n* = 5) were cultured alone or with PMA/ionomycin in the presence of GolgiStop. After 4 hours of culture, cells were stained with dead cell exclusion dye as well as antibodies to detect surface CD3, CD4, CD8, PD-1, TIGIT, intracellular IFN-γ, and IL-2 and analyzed using flow cytometry. (**A**) Proportion of CD8^+^ and CD4^+^ T cells secreting IFN-γ and IL-2. (**B**) IFN-γ secretion by cells expressing PD-1 and/or TIGIT. Figure shows a representative plot from patient with AML. All graphs show mean ± SEM. *P < 0.05 by Mann-Whitney *U* test with Bonferroni’s correction for multiple comparisons.

Chronic antigen stimulation in cancer is associated with the emergence of T cell exhaustion and resultant dysfunction (18). Therefore, we analyzed the presence of several immune activating and inhibitory checkpoints on the surface of these cells. Among the agonistic molecules studied, the expression of 4-1BB was significantly increased in the CD8^+^ T cells from leukemic patients (Figure 1D), while the proportion of ICOS- and OX40-expressing T cells were comparable (Supplemental Figure 1, E and F). T cells within the leukemic BM also expressed higher levels of several inhibitory immune checkpoints. Among T cells infiltrating B-ALL, both CD4^+^ and CD8^+^ T cells expressed higher levels of TIGIT, LAG3, and PD-1, compared with HD (Figure 1E). Among T cells infiltrating AML, both CD4^+^ and CD8^+^ T cells expressed higher levels of LAG3 and PD-1 (Figure 1F). The proportion of T cells coexpressing more than 1 inhibitory checkpoint (PD-1, LAG3, and TIGIT) was increased in the leukemic marrow (Supplemental Figure 1G). Expression of TIM3 and CTLA4 was not different in T cells from either leukemic cohort (Supplemental Figure 1, E and F). Expression of inhibitory checkpoints is often associated with emergence of T cell exhaustion or dysfunction, leading to a decreased ability to secrete cytokines (19). Therefore, we analyzed cytokine production in these T cells using flow cytometry. Both CD4^+^ and CD8^+^ T cells infiltrating AML BM had reduced capacity for IFN-γ secretion (Figure 2A). CD4^+^ T cells from AML BM had reduced capacity for IL-2 secretion, as well (Figure 2A). There were no differences in IL-4 or IL-17 production by these T cells (Supplemental Figure 1H). Importantly, even T cells expressing PD-1 or TIGIT retain capacity for cytokine production, indicating that they are not fully exhausted but rather dysfunctional, consistent with phenotypes described in some solid tumors (20) (Figure 2B). Together, these data illustrate that T cells infiltrating pediatric AML and B-ALL express increased but variable patterns of inhibitory checkpoints (particularly TIGIT, in the case of B-ALL, and LAG3, in the case of AML), and also show evidence of T cell dysfunction but not complete exhaustion.

**Figure 3:**
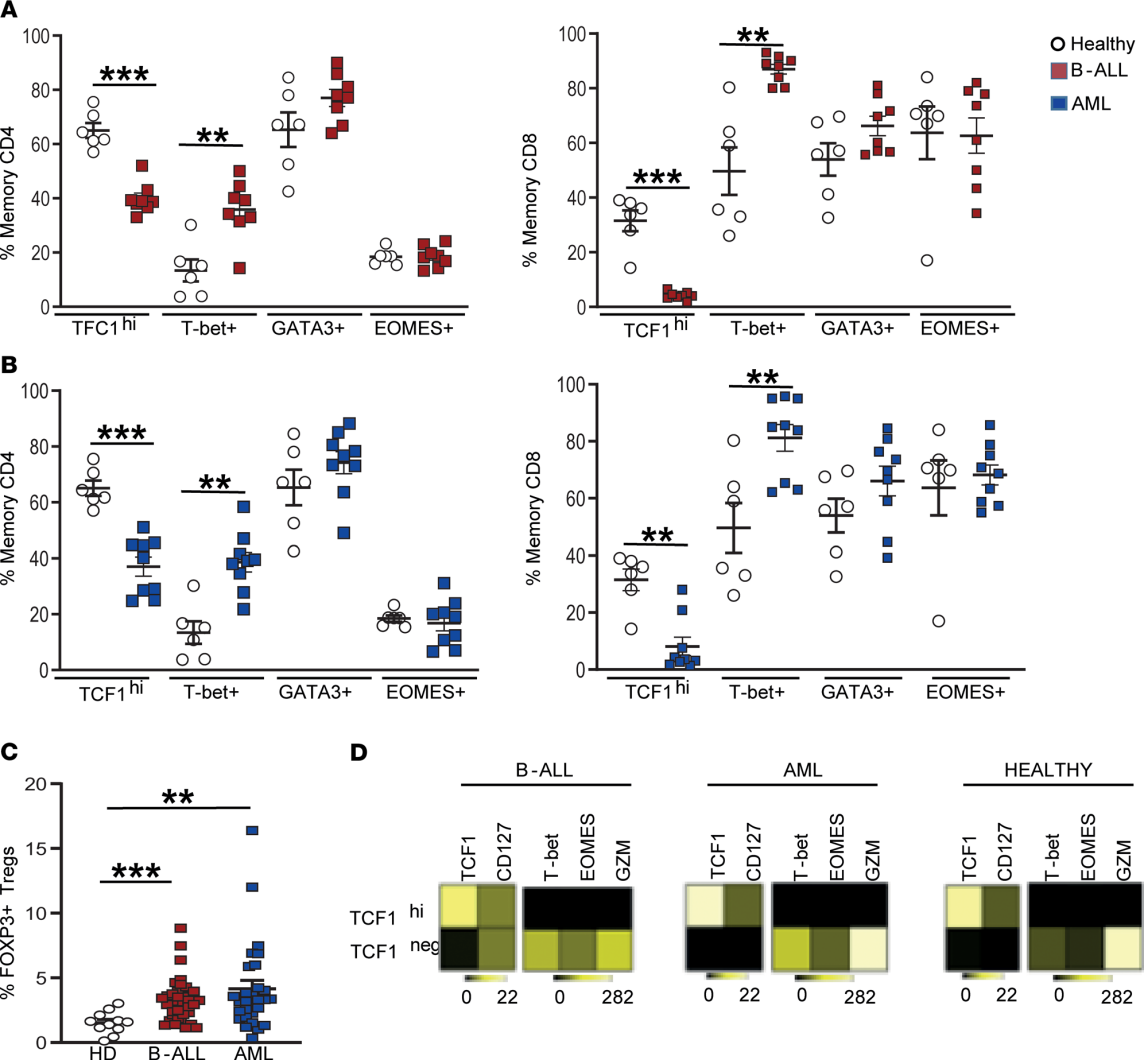
Changes in BM T cell transcription factors in children with B-ALL and AML at diagnosis. BMMNCs from B-ALL (*n* = 13), AML (*n* = 17), or HD (*n* = 5) were cultured alone or with PMA/ionomycin in the presence of GolgiStop. After 4 hours of culture, cells were stained with dead cell exclusion dye as well as antibodies to detect surface CD3, CD4, CD8, PD-1, TIGIT, intracellular IFN-γ, and IL-2 and analyzed using flow cytometry. (**A**) Expression of TCF1, T-bet, GATA3, and EOMES transcription factors in memory CD4^+^ (left) and CD8^+^ (right) T cells in BMMNCs from HD and patients with B-ALL. (**B**) Expression of TCF1, T-bet, GATA3, and EOMES transcription factors in memory CD4^+^ (left) and CD8^+^ (right) T cells in BMMNCs from HD and patients with AML. (**C**) Tregs (CD3^+^CD4^+^CD25^+^CD127^–^FOXP3^hi^) as percentage of total T cells in BM from HD (*n* = 11) and patients with B-ALL (*n* = 36) and AML (*n* = 28). (**D**) Heatmaps showing characteristics of the TCF1^hi^ and TCF1^–^ CD8^+^ memory T cells from HD, B-ALL, and AML. All graphs show mean ± SEM. **P < 0.01, ***P < 0.001 by Mann-Whitney *U* test with Bonferroni’s correction for multiple comparisons.

Emergence of terminal effector T cell differentiation, as well as T cell dysfunction, is regulated by functional properties of key transcription factors (19). Therefore, in order to gain further insights into the possible mechanisms underlying changes observed earlier, we analyzed the expression of transcription factors known to regulate T cell differentiation and exhaustion (TCF1, T-bet, EOMES, and GATA3) using mass cytometry. Of these, the expression of TCF1 and T-bet were significantly altered in leukemic marrow–infiltrating T cells compared with HD, while the expression of other factors such as GATA3 and EOMES was not different (Figure 3, A and B). Both AML and B-ALL T cells were also associated with a higher proportion of FOXP3-expressing Tregs (CD3^+^CD4^+^CD25^+^CD127^–^FOXP3^hi^ cells; Figure 3C). Observed differences in TCF1-expressing cells were of interest due to its emerging role in maintaining long-term memory T cells and preventing the attrition of exhausted T cells (21–23). Prior studies have shown that TCF1 expression in human memory T cells follows a distinct gradient, with TCF1^hi^T-bet^lo^ cells linked to stem-like features, with greater capacity for self-renewal and asymmetric cell division (24, 25). Using a similar gating strategy, we observed that TCF1^hi^ memory T cells in the BM have a distinct phenotype with higher expression of CD127 and reduced expression of lytic genes such as granzyme B (Figure 3D). Thus, CD8^+^ memory T cells infiltrating leukemic BM are deficient in phenotypes associated with stem-like memory (18).

### Changes in NK cells.

**Figure 4:**
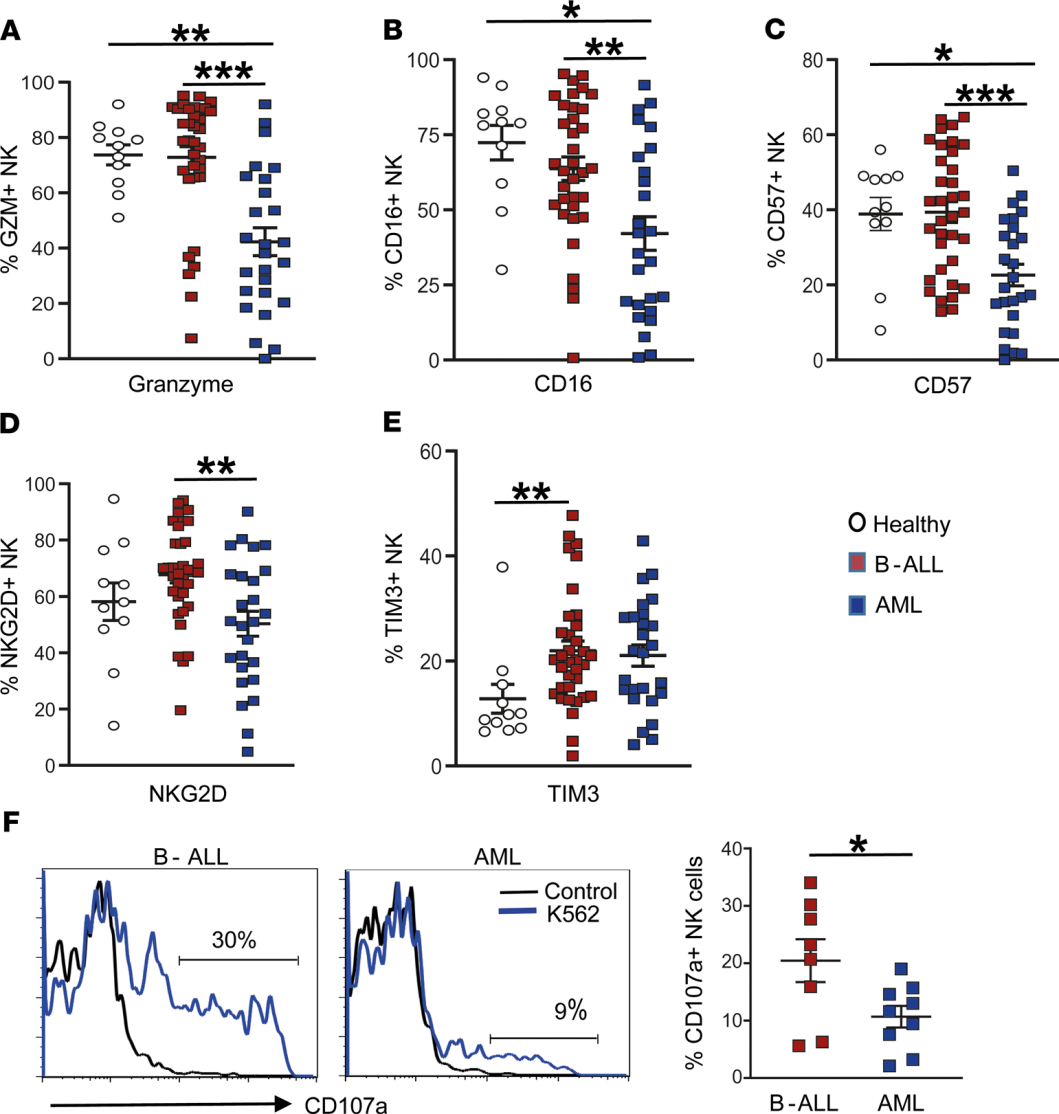
Changes in BM NK cells. (**A–E**) Expression of granzyme (GZM) (**A**), CD16 (**B**), CD57 (**C**), NKG2D (**D**), and TIM3 (**E**) on BM NK cells from HD (*n* = 11), as well as patients with B-ALL (*n* = 35) and AML (*n* = 26). (**F**) Histograms show change in surface expression of CD107a as a marker of NK cell degranulation upon NK cell culture alone (control) or with K562 cells. Left panels are representative patients, and the graph on the right shows data from several patients (B-ALL, *n* = 8; AML, *n* = 9). All graphs show mean ± SEM. *P < 0.05, **P < 0.01, ***P < 0.001 by Mann-Whitney *U* test with Bonferroni’s correction for multiple comparisons.

Next, we analyzed changes in CD3^–^CD56^+^ NK cells infiltrating the leukemic BM. Changes in NK phenotype were most evident in AML, with significant declines in granzyme (Figure 4A), CD16 (Figure 4B), CD57 (Figure 4C), and NKG2D (Figure 4D). In addition, there was a modest increase in the expression of TIM3 in NK cells from leukemia patients (Figure 4E). In order to further analyze the functional properties of NK cells infiltrating leukemic marrow, we compared the capacity of NK cells to express CD107a as a marker of degranulation upon coculture with K562 cells as NK targets. NK cells from AML patients exhibited significantly reduced capacity for degranulation compared with those from B-ALL, consistent with other phenotypic changes (Figure 4F). Together, these data show that NK cells from children with AML exhibit functional defects.

### Discriminating immune microenvironment in leukemias using SPADE analysis.

While the analyses focusing on individual cell types discussed earlier identified significant differences between immune cells infiltrating the BM of both B-ALL and AML patients compared with HD, we used an automated spanning tree progression analysis for density normalized events (SPADE) algorithm (26–28) integrating data from all parameters in an unbiased fashion (Supplemental Figure 2A). Application of classifiers built on this algorithm revealed that T cells from HD, AML, or B-ALL BM differed based on their phenotypic properties (Supplemental Figure 2B). These data also confirm several differences noted in earlier analyses, such as higher expression of TIGIT in B-ALL CD4^+^ T cells compared with AML and HD and higher expression of T-bet in CD8^+^ memory T cells from patients with B-ALL and AML compared with HD (Supplemental Figure 2C).

### Immune features associated with disease risk.

**Figure 5:**
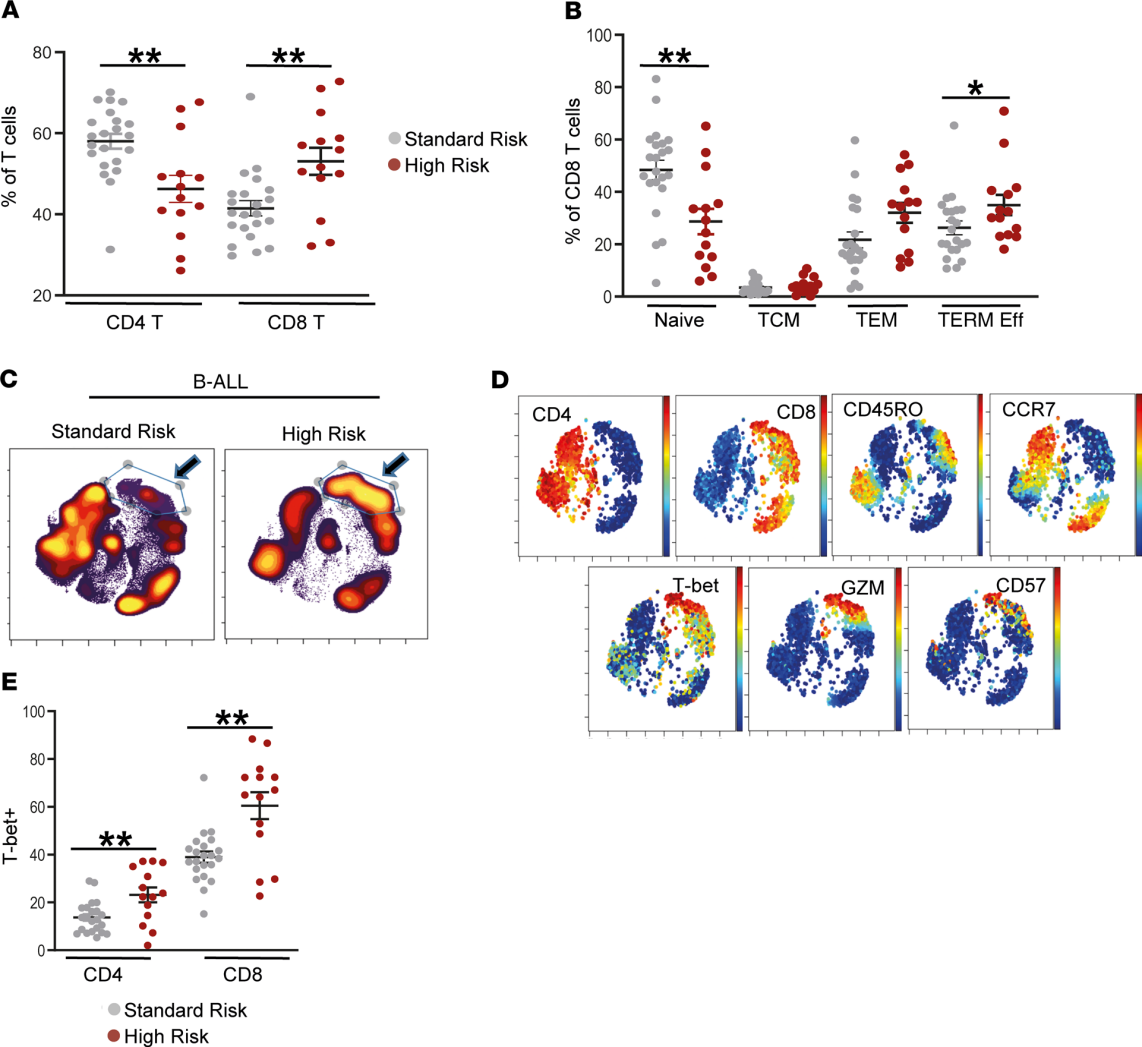
Immune correlates of clinical disease risk in B-ALL. BMMNCs from patients with standard-risk (*n* = 22) and high-risk (*n* = 16) B-ALL were characterized using single cell mass cytometry. (**A**) Distribution of CD4^+^ and CD8^+^ T cells by risk status. (**B**) Frequency of naive, central memory (TCM), effector memory (TEM), and terminal effector (TERM Eff) CD8^+^ T cells by risk status. (**C**) viSNE density plots for patient groups (standard risk, *n* = 17; high risk, *n* = 9) visualized by concatenating FCS files for patients within each risk group. (**D**) viSNE plots showing expression of CD4, CD8, CCR7, CD45RO, T-bet, granzyme (GZM), and CD57 from a representative patient. (**E**) Expression of T-bet in memory CD4^+^ and CD8^+^ T cells from B-ALL patients with standard-risk or high-risk disease. All graphs show mean ± SEM. *P < 0.05, **P < 0.01 by Mann-Whitney *U* test.

In order to test whether immune changes could, in part, underlie the biologic basis of the current clinical risk classification, we analyzed changes in immune cells in the context of current clinical disease risk models (see Supplemental Table 1 for definitions of disease risk) (5). Compared with standard-risk (SR) B-ALL patients, those with high-risk (HR) disease had higher proportion of CD8^+^ T cells and lower proportion of CD4^+^ T cells (Figure 5A). HR B-ALL was also characterized by greater decline in naive CD8^+^ T cells, as well as an increase in terminal effector CD8^+^ T cells in the BM compared with SR patients (Figure 5B). This is also evident when mass cytometry data from all HR or SR patients are concatenated and analyzed together. The cluster of T cells enriched in HR-ALL has a phenotype of CD45RO^+/–^ Granzyme^+^ CD57^+/–^ T-bet^+^ CCR7^–^ CD8^+^ T cells (Figure 5, C–E).

**Figure 6:**
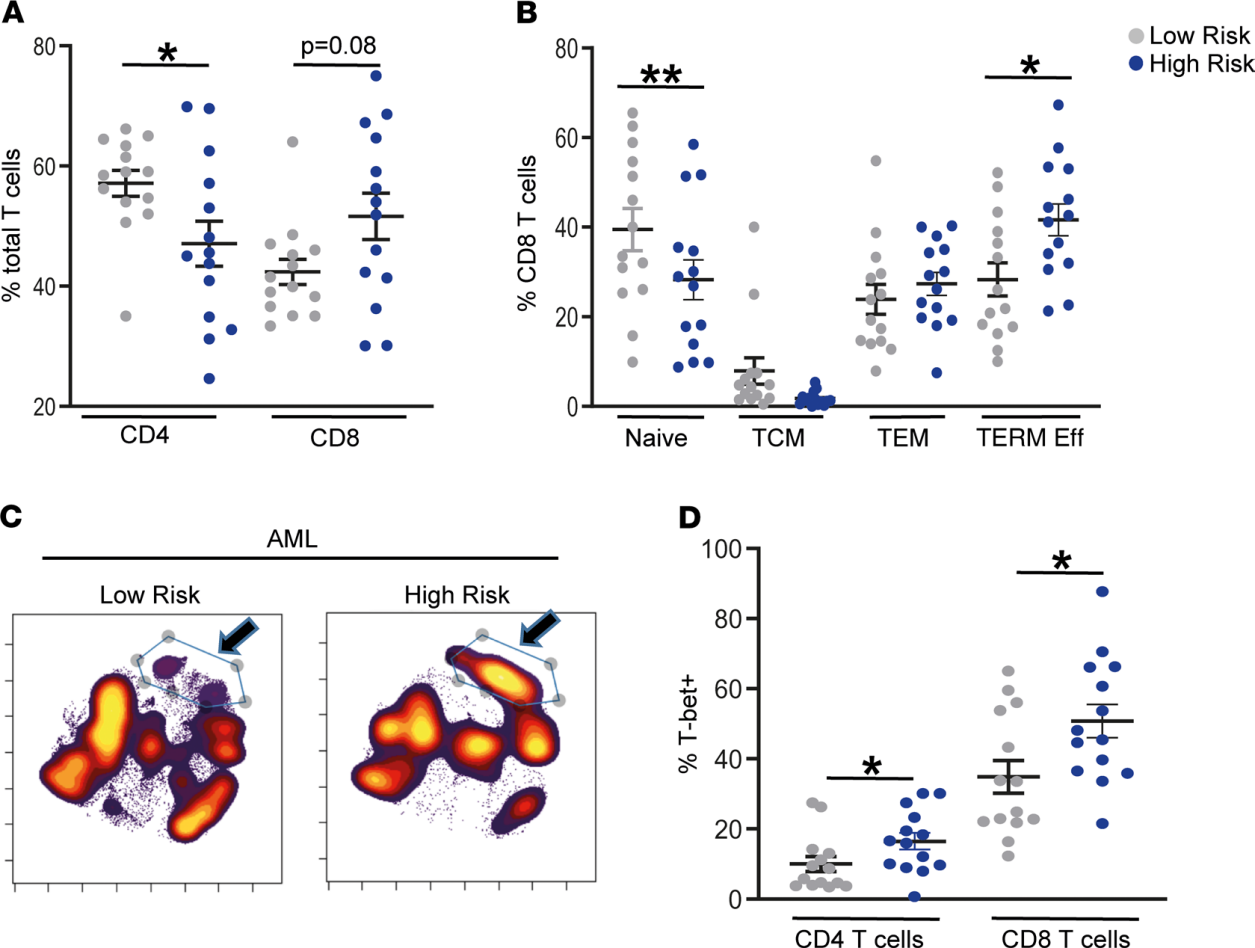
Immune correlates of clinical disease risk in AML. BMMNCs from patients with low-risk (*n* = 14) and high-risk (*n* = 14) AML were characterized using single cell mass cytometry. (**A**) Distribution of CD4^+^ and CD8^+^ T cells by risk status. (**B**) Frequency of naive, central memory (TCM), effector memory (TEM), and terminal effector (TERM Eff) CD8^+^ T cells by risk status. (**C**) viSNE density plots for patient groups (low risk, *n* = 10; high risk, *n* = 10) visualized by concatenating FCS files for all patients within each disease risk group. (**D**) Expression of T-bet in memory CD4^+^ and CD8^+^ T cells from AML patients with low-risk or high-risk disease. All graphs show mean ± SEM. *P < 0.05, **P < 0.01 by Mann-Whitney *U* test.

As with B-ALL, HR AML was also characterized by greater accumulation of terminal effector T cells, concurrent with decline in naive T cells (Figure 6, A and B). This is also evident when data from all HR or SR AML patients are concatenated and analyzed together (Figure 6C). The phenotype of the expanded population in HR AML is similar to that in HR B-ALL and includes an increase in T-bet^+^CD57^+^Granzyme^+^CD27^lo^ memory T cells (Figure 6D). Together, these data suggest that progressive effector differentiation of BM T cells may be a common feature of HR clinical phenotype in childhood leukemia.

### Immune subtypes of childhood leukemia.

**Figure 7:**
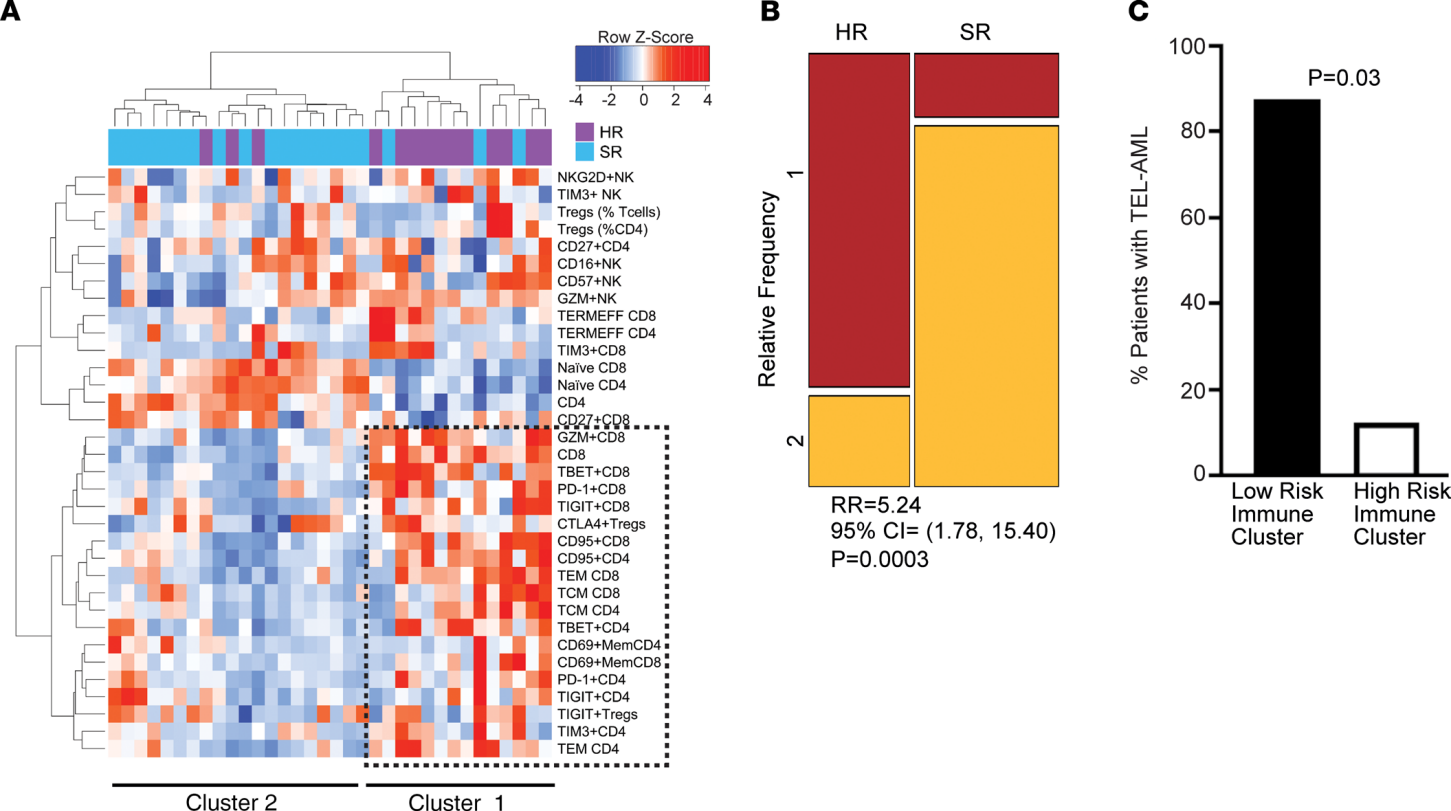
Distinct immune clusters associate with disease risk and outcome in childhood B-ALL. BMMNCs from patients with standard-risk (SR, *n* = 22) and high-risk (HR, *n* = 16) B-ALL were characterized using single cell mass cytometry. (**A**) Hierarchical cluster analysis based on immune markers in B-ALL. (**B**) Mosaic plot showing distribution of HR and SR B-ALL patients in the 2 clusters (relative frequency in cluster 1 is in red and cluster 2 is in yellow). *P* value corresponds to Wald’s test. (**C**) Bar graph showing distribution of TEL-AML^+^ B-ALL patients in the immune clusters. *P* value corresponds to Wald’s test.

Recent studies in some adult tumors have shown that properties of infiltrating immune cells can be used to identify immune subtypes of tumors that may be amenable to distinct immune-based interventions (29). To date, classification of childhood leukemia has been based largely on genetic changes in tumor cells, without attention to host response. Our finding that immune activation is a common feature of leukemia raised the possibility that immune changes in the tumor microenvironment may identify distinct clinically relevant “immune subtypes.” Hierarchical cluster analysis using mass cytometry variables discussed above identified 2 broad immune subtypes of B-ALL. Immune cluster 1 with greater CD8^+^ effector cell differentiation was enriched in patients with HR disease (*P* = 0.0003; Figure 7, A and B). In contrast, B-ALL patients with favorable cytogenetics (TEL-AML1 [ETV6-RUNX1] fusion) were enriched in immune cluster 2 (*P* = 0.03; Figure 7C).

**Figure 8:**
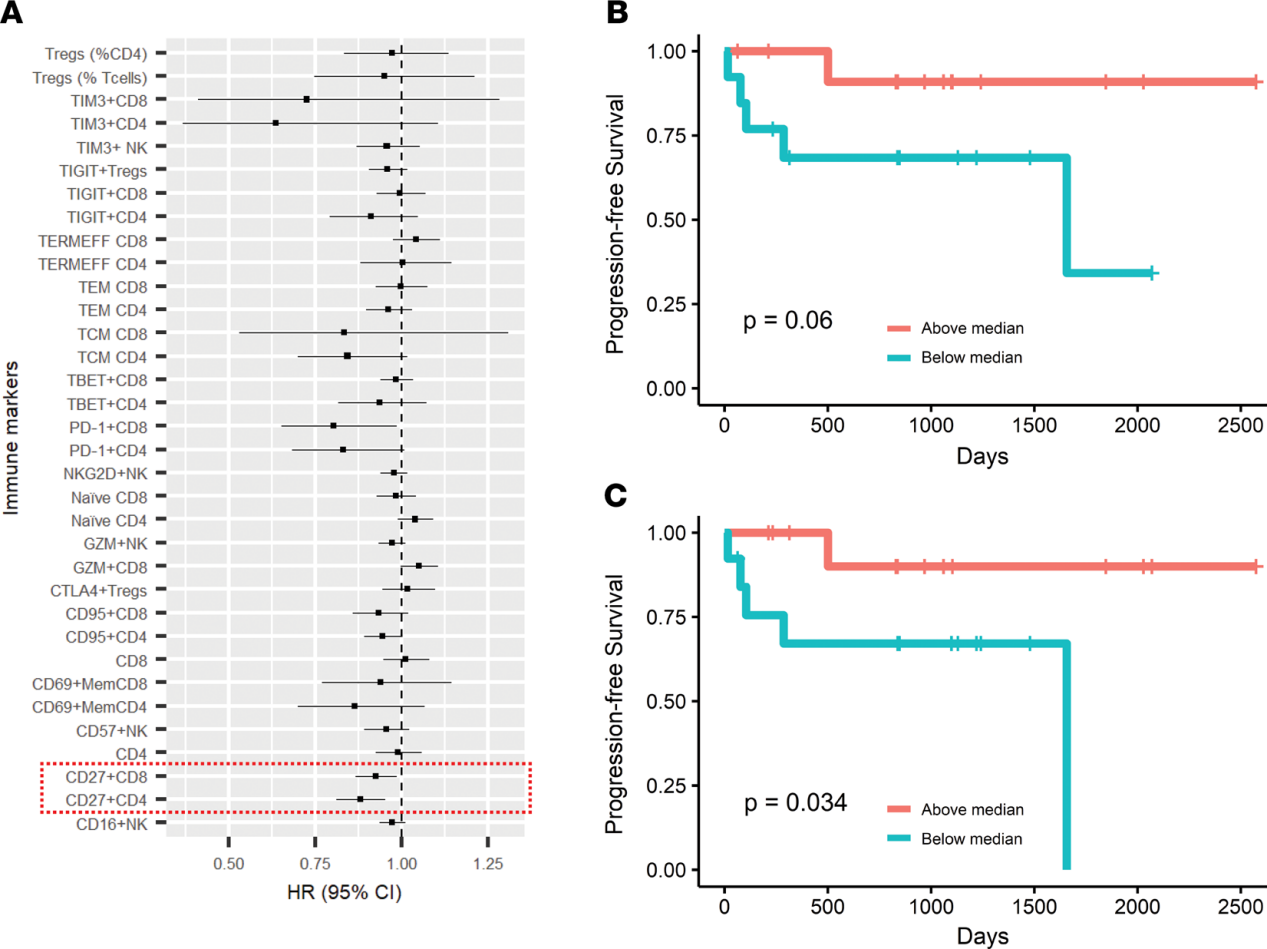
Distinct immune clusters associate with disease risk and outcome in childhood AML. BMMNCs from patients with AML (*n* = 28) were characterized using single cell mass cytometry. (**A**) Forest plot showing Cox regression analysis of immune markers with progression-free survival in AML. (**B** and **C**) Progression-free survival of AML patients with above- versus below-median proportions of CD27^+^CD4^+^ T cells of total CD4^+^ T cells (**B**) and CD27^+^CD8^+^ T cells (**C**) of total CD8^+^ T cells. *P* values correspond to log-rank test.

Similar cluster analysis also identified 2 broad immune subtypes of AML patients (Supplemental Figure 3), again differentiated based on the proportion of T cells with terminal effector differentiation. However, in this setting, patients with clinical HR AML were distributed in both clusters. We performed multivariate Cox regression analysis to assess the association between immune markers and progression-free survival in AML patients (Figure 8A). Cox regression analysis revealed that lower proportions of CD27^+^CD4^+^ T cells (*P* = 0.06) and CD27^+^CD8^+^ T cells (*P* = 0.03) were associated with significantly shorter progression-free survival (Figure 8, B and C).

### Validation of mass cytometry data by single cell transcriptomics.

**Figure 9:**
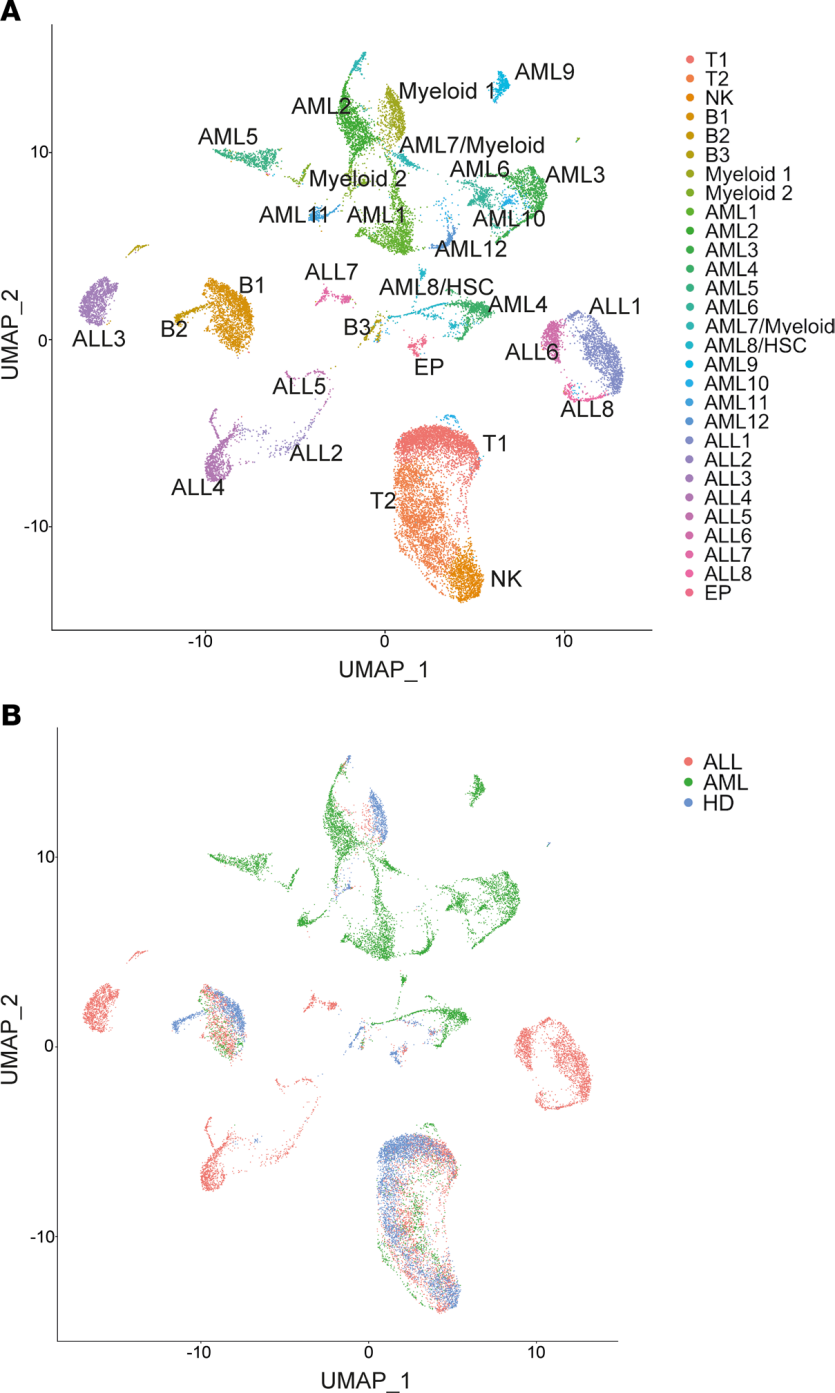
Single cell transcriptome analysis of BM mononuclear cells in childhood B-ALL and AML. A total of 24,081 BM mononuclear immune cells from 19 samples (4 healthy donor, 8 AML, and 7 B-ALL) was characterized using single cell mRNA sequencing. (**A**) Uniform manifold approximation and projection (UMAP) plot with 29 distinct cell populations determined by unsupervised clustering (T, T cells; NK, NK cells; B, B cells; EP, erythroid progenitor cells; HSC, hematopoietic stem cells). (**B**) UMAP plot distinguishing cells by disease state.

**Figure 10:**
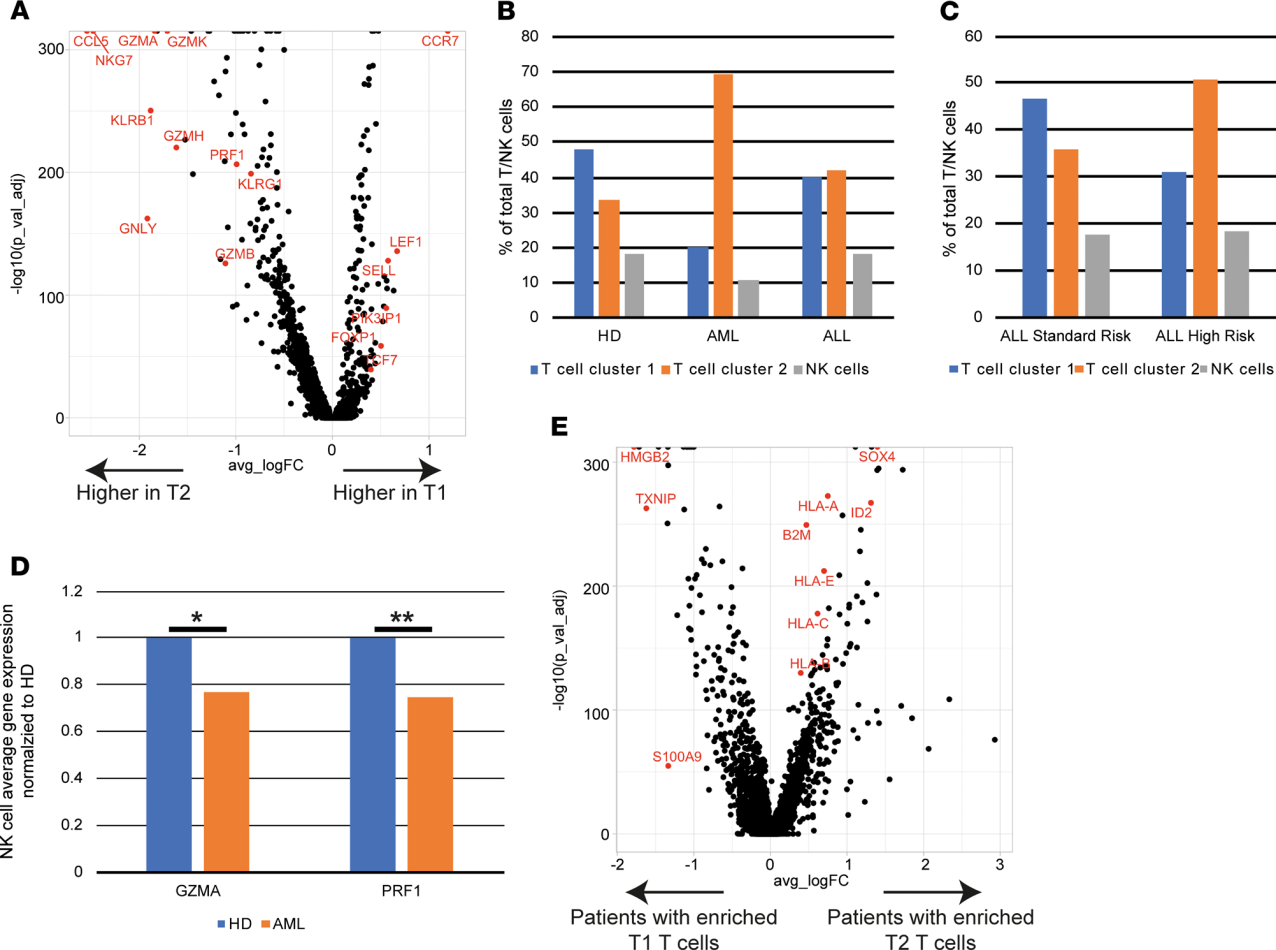
Single cell transcriptome analysis of BM mononuclear cells in childhood B-ALL and AML. A total of 24,081 BM mononuclear immune cells from 19 samples (4 healthy donor, 8 AML, and 7 B-ALL) were characterized using single cell mRNA sequencing. (**A**) Volcano plot of differential gene expression between T cell cluster T1 (naive/stem like enriched in HD) and T cell cluster T2 (effector-like enriched in malignancy). Adjusted *P* value (p_val_adj) corresponds to Wilcoxon rank-sum test with Bonferroni’s correction. Positive log-fold change (avg_logFC) corresponds to higher expression in T1 relative to T2. (**B**) Cells in each T/NK cell cluster as a percentage of total T/NK cells by disease state. (**C**) Distribution of T cells in cluster T1 and T2 in B-ALL patients based on NCI disease risk (standard risk; high risk). (**D**) Expression of granzyme (GZMA) and perforin (PRF1) in NK cells from healthy donors (HD) and AML patients (AML). *P < 0.01, **P < 0.0001 by Wilcoxon rank-sum test with Bonferroni’s correction. (**E**) Volcano plot of genes differently regulated between tumor cells from patients with T cells enriched for naive/stem like phenotype (T1 T cells) versus tumor cells from B-ALL patients with BM enriched for terminally differentiated effector T cells or T2 cluster.

In order to further validate the changes observed with mass cytometry as described above, we also analyzed some of these samples by scRNA-Seq. In order to maintain the focus on the immune compartment, BMMNCs were first enriched for immune cells by flow sorting based on CD45^hi^ expression. After excluding low-quality cells, k-nearest neighbor clustering of 24,081 cells created a detailed map composed of 29 transcriptionally distinct subpopulations, including 8 immune clusters and 20 tumor cell clusters (Figure 9, A and B). Markers defining the clusters are shown in Supplemental Figure 4. Two broad clusters of T cells (T1 and T2) were identified. T2 cluster was characterized by greater effector differentiation and increased expression of genes, including *NKG7*, granzyme, perforin, granulysin, and *KLRG1*, and T1 cluster was enriched for genes such as *CCR7*, L selectin, and *TCF7*, suggesting that it contained more naive/stem-like T cells (Figure 10A). Pathway analysis of differentially expressed genes between these clusters revealed enrichment of pathways associated with T cell activation/effector differentiation in the T2 cluster and enrichment of pathways associated with naive T cells in the T1 cluster (Supplemental Figure 4B). Consistent with prior mass cytometry data (Figure 1) showing increase in effector T cells in leukemia patients compared with HD, scRNA-Seq also revealed a greater proportion of T cells in the T2 (effector like) cluster in AML and B-ALL BM (Figure 10B). Similarly, consistent with prior mass cytometry data showing greater effector T differentiation in HR-ALL, scRNA-Seq also identified enrichment of the T2 cluster with greater effector differentiation in the HR B-ALL cohort (Figure 10C). In addition to T cells, scRNA-Seq analysis also validated prior mass cytometry data showing a decrease in granzyme expression in NK cells from AML patients (Figure 10D). Together, these data provide validation of the mass cytometry findings.

Because mass cytometry revealed that patients with favorable cytogenetics were associated with less effector T cell differentiation (Figure 7, A and C), we used scRNA-Seq data to compare transcriptomes of tumor cells between patients with enrichment of T1 (naive like) or T2 (effector like) T cell clusters. Interestingly, this revealed that tumors with increased effector T cells were enriched for higher expression of stem/progenitor cell–associated genes such as *SOX4* and *ID2*. These tumors also had higher expression of immune genes (such as HLA genes [both *MHC-I* and *HLA-E*] and β-2 microglobulin) (Figure 10E). Therefore, HR immune signatures may be linked to properties of tumor cells such as the presence of stem-like genes.

## Discussion

In this paper, we have used complementary single cell tools to study the immune landscape of the tumor microenvironment in childhood leukemias. Our data show that immune activation and subsequent dysfunction in both innate and adaptive immunity are common features of childhood leukemias. These data have broad biologic implications for cancer immunology, since pediatric leukemias have typically been a prototype of tumors with relatively low mutational burden and therefore fewer mutation-derived neoantigens. Contrary to this dogma, our data suggest that childhood leukemias are commonly immunogenic and lead to significant alterations in the BM microenvironment.

Although pediatric leukemias, by definition, have a shorter natural history compared with many adult tumors, T cells in the BM of these patients already exhibit markers of chronic immune activation and exhaustion/dysfunction recently described in adult tumors (18). Recent studies have shown that maintaining protective immunity in the setting of chronic antigenic stimulation such as chronic viral infections may depend on a subset of TCF1^hi^ memory stem-like T cells, which prevent attrition of exhausted T cell clones over time (23, 25, 30, 31). Our finding that this compartment is markedly depleted in childhood leukemias provides a potential explanation for the inability of the immune system to naturally control these tumors at diagnosis in spite of an apparently abundant immune response to neoantigens (13). These data also show that the hierarchy of T cell exhaustion is not just a feature of adult tumors as described to date (25, 32), but also is present in tumors in children who have a much lower mutational burden. Since TCF1^+^ cells have been implicated in the proliferative burst following immune checkpoint blockade (31), these data may also provide an explanation for the apparent lack of clinical activity of PD-1 pathway inhibitors in childhood leukemia.

Our data identify similarities as well as distinct differences in the immune microenvironment between B-ALL and AML that may be clinically relevant and carry translational potential. For example, our data suggest that dysfunction of NK cells may be more prominent in AML than in B-ALL and may contribute to greater immune paresis and susceptibility to infections in these patients. Evaluation of immune therapies in childhood leukemias has been limited to T cell redirection strategies, with limited evaluation of immune checkpoints mostly limited to the PD-1 pathway (33). Our finding that the expression of TIGIT and LAG3 is increased in marrow-infiltrating T cells supports specific evaluation of these targets, already under active investigation in adult cancers, in pediatric leukemia (34).

It is of interest that clinical HR leukemias (of either subtype) are associated with depletion of naive T cells and greater effector CD8^+^ T cell differentiation — particularly, accumulation of terminal effector T cells. Properties of the host response may therefore contribute to the biologic basis of the observed differences in clinical risk. The degree to which the increased effector T cell differentiation and consequent enrichment of terminal effectors or depletion of stem-like TCF1-expressing memory T cells is reversible is not known, but properties of endogenous T cell response may have a major impact on the durability of responses to immune therapies, including following T cell redirection in children treated with CAR-T cells (22).

Strengths of this analysis are that it is based on high-dimensional single cell proteomic data in the BM and focused on immune cells, with both cell surface and intracellular markers and not just limited to single cell transcriptomic analyses. Potential weaknesses include lack of data on survival in B-ALL cohort, since many of the B-ALL samples were collected as a part of a Children’s Oncology Group (COG; www.childrensoncologygroup.org) biology study lacking such data. Due to ethical considerations and ability to provide consent, the HD were limited to young adults instead of children. However, older age of HD is unlikely to be a major factor, as their immune cells were more similar to younger SR B-ALL patients (median age 4.5 years) compared with older HR patients. While the focus in this manuscript is on immune cells, other (nonimmune) cells in the tumor microenvironment may also be important. Another potential limitation of this study is the use of cryopreserved specimens. While this facilitated the concurrent analysis of specimens from leukemia patients and healthy controls together to help reduce the impact of interassay variance, use of thawed specimens may not measure a representative sample of the in vivo setting, as it can particularly impact certain populations such as granulocytes.

The mechanisms underlying altered T cell differentiation in leukemia deserve further study but may depend, in part, on genomic features of tumor cells (35). In this regard, it is of interest that the HR immune signatures were associated with expression of genes such as *SOX4* and *ID2* that are associated with a less differentiated phenotype of leukemic cells (36, 37). Interestingly, expression of these genes on tumor cells is also associated with higher expression of HLA genes, suggesting that these tumors may be less subject to immune pressure under natural settings. Understanding the mechanisms of immune evasion by these cells and targeting the pathways involved may be essential to maintaining immune control of tumors (38, 39).

## Methods

### Patients and samples.

BM aspirates obtained at the time of diagnosis (B-ALL, *n* = 36; AML, *n* = 28) were used for the described studies. Samples were obtained from children, adolescents, and young adults from the COG or local institutions via informed consent on IRB-approved protocols in accordance with the Declaration of Helsinki. For COG and Aflac biorepository, samples were requested with a diagnosis of either SR or HR B-ALL or low-risk and HR AML. No other criteria were prespecified for sample selection. Yale University samples used in our study were obtained sequentially. Fresh BM aspirates from HD (*n* = 11) were purchased through All Cells Inc. Due to ethical considerations and ability to provide informed consent, BM aspirates from HD were obtained from donors aged > 18 years but limited to younger adults (median age 24 years). BMMNCs were obtained by ficoll density gradient centrifugation. All samples were thawed and processed similarly to reduce assay variability. The viability of the samples assessed by single cell mass cytometry was > 80%. The mean (±SD) BM blast percentages were 77% (±17%) for B-ALL and 66% (±13%) for AML.

### Mass cytometry.

Thawed BM suspensions were stained with two 37-marker panels using metal conjugated antibodies according to manufacturer-suggested concentrations (Fluidigm) as previously described (25, 40). Data on specific antibodies and clones are provided in Supplemental Table 2. Cells were fixed, permeabilized, and washed according to manufacturer’s cell surface and nuclear antigen staining protocol (Fluidigm). After antibody staining, cells were incubated with intercalator solution, washed, mixed with EQ Four Element Calibration Beads (catalog 201078), and acquired with mass cytometer (all reagents from Fluidigm). We targeted minimum 50,000 events, and > 100,000 events were collected for > 90% of the samples. The median number of events collected per sample was 260,000. Gating and data analysis were performed with Cytobank (https://www.cytobank.org/) or SPADE. Intact viable cells were identified using cisplatin and iridium intercalator according to manufacturer-suggested concentrations (Fluidigm). viSNE analysis was performed with Cytobank. Proportional sampling of cells from each patient was done before viSNE analysis and run simultaneously to visualize patients’ viSNE plots. viSNE plots for patient groups were visualized by concatenating FCS files for all patients within each health risk group (Cytobank).

### SPADE analysis.

A total of 51 FCS files were generated to profile 41 protein markers across 51 subjects, including 27 B-ALL patients, 19 AML patients, and 5 HD. The original FCS files were input into FlowJo software and exported after gating on viable cells based on DNA labeling with iridium (Ir191/193), cell length, and cisplatin. The exported FCS files were transferred into the SPADE software (spanning-tree progression analysis of density-normalized events) for analysis (28). The data were transformed using hyperbolic inverse sine transformation, with the cofactor being 5. SPADE was used to analyze all FCS files jointly. More specifically, SPADE performed density-dependent downsampling on individual samples separately, pooled the downsampled cells, performed clustering, constructed a tree structure, and performed upsampling to compute summary statistics based on all cells in the data. The SPADE tree represented all cell phenotypes that existed in at least 1 of the samples. The SPADE tree (shown in Supplemental Figure 2), colored by several of the measured surface protein markers, collectively shows that different regions of the tree corresponded to different marker combinations. A semiautomated method was used to partition the tree into pieces based on the colored versions of the SPADE tree and resulting in pieces with biologically interpretable annotations (26). Two example annotations are shown in Supplemental Figure 2. For each annotated phenotype, median intensity of the marker expression was computed for each protein marker for each sample and visualized in heatmaps to illustrate the differences of protein expression combinations in annotated phenotypes on the SPADE tree.

The annotated SPADE tree is used to derive features for representing individual patients. For each patient, the features include average expression of each protein marker in each annotated phenotype, as well as the cell percentage frequency of each annotated phenotype (41). The feature-patient matrix is used as input for random forest analysis with 5-fold cross-validation, which evaluates whether various patient groups (ALL, AML, and HD) can be accurately classified based on the SPADE-derived features. Using the top 20 features identified by random forest, Euclidean distance is used to compute pairwise patient-to-patient similarities. Heatmap visualization of the patient-to-patient similarities is able to reveal subgroups of patients in the data.

### Functional studies.

BMMNCs were thawed and rested overnight before performing functional assays, including detection of intracellular cytokines and NK degranulation assays, as previously described (42).

Analysis of intracellular cytokines T and NK cells was performed as previously described (43). For cytokine detection, cells were stimulated with phorbol 12-myristate 13-acetate and ionomycin, both at a 500 ng/mL concentration for 5 hours at 37°C in the presence of protein transport inhibitor BD Golgi Stop (0.7 μL/mL). Cells were labeled with dead cell exclusion dye (Thermo Fisher Scientific, catalog L23101), anti-CD3 (clone SP34-2, BD Biosciences, catalog 552851), -CD4 (MT477, BD Biosciences, catalog 742738), -CD8 (SK1, BD Biosciences, catalog 560179), -CD56 (clone NCAM16.2, BD Biosciences, catalog 564447), –PD-1 (clone J105, eBioscience, catalog 25-2799-42), and -TIGIT (MBSA43, eBioscience, catalog 17-9500-42), as well as anti–IFN-γ (clone B27, BD Biosciences, catalog 562392), –IL-2 (clone MQ1-17H12, BioLegend, catalog 500320), –IL-4 (8D4-8, BD Biosciences, catalog 554516), and –IL-17 (clone BL168, BioLegend, catalog 512322) antibodies. Data were analyzed using FlowJo software.

For NK functional studies, BMMNCs from AML (*n* = 9) and B-ALL (*n* = 8) patients were cultured alone or with K562 cells in an effector to target ratio of 1:10. Cells were stained for CD107a (clone H4A3, BD Biosciences, catalog 560664) for 4 hours in the presence of GolgiStop (0.7 μg/mL; BD Biosciences) to detect degranulation. Following incubation, cells were stained for CD3, CD8, and CD56 and analyzed using flow cytometry.

### scRNA-Seq and data analysis.

BMMNCs were thawed for single cell mRNA sequencing and sorted to enrich immune cells based on dead cell exclusion dye and expression of CD45. Barcoded libraries were prepared using the manufacturer’s (10x Genomics) version 2 protocol and sequenced with Illumina HiSeq. Reads were aligned, filtered, deduplicated, and converted into a digital count matrix using Cell Ranger 1.2 (10x Genomics). Additional quality control and data analysis were performed using the Seurat v3 package in R (44). Cells with fewer than 200 unique sequenced genes or more than 10% mitochondrial genes were removed to exclude poorly sequenced cells, and cells with more than 7000 unique sequenced genes or 70,000 sequenced features were removed to exclude potential doublets. Gene expression for each cell was log normalized to total expression per cell using the Seurat NormalizeData function. A total of 2000 highly variable genes across all cells were selected using variance-stabilizing transformation with the Seurat FindVariableFeatures function. Expression of each gene was shifted to achieve a mean of 0 across all cells and then scaled such that the variance across all cells was 1, using the ScaleData function. Principal component analysis (PCA) was then performed on this scaled data using the 2000 previously identified highly variable genes. The top 20 principal components (PCs) were then used for clustering. First, a k-nearest neighbor graph was constructed in this 20 PC space, and the edge weights were refined based the Jaccard similarity using the FindNeighbors function. Then, the Louvain community algorithm was used to optimize modularity by iteratively grouping cells until optimal clustering was reached using the FindClusters function with a resolution of 0.6. For visualization of results, uniform manifold approximation and projection (UMAP) was performed using the top 20 PCs to project the data into a 2-dimensional space. To determine cluster identity, differential gene expression was calculated between each cluster and all others. Significantly differentially expressed genes were identified by the Wilcoxon rank-sum test with a Bonferroni’s correction (*P* < 0.05). Cluster identity was determined by inspection of canonical marker genes (e.g., CD3 for T cells) and confirmed by comparison with documented gene expression profiles of immune cells with SingleR (45). The final data set consisted of 24,081 cells from 19 samples (HD, *n* = 4; B-ALL, *n* = 7; and AML, *n* = 8). Pathway analysis of significantly differentially expressed genes (Wilcoxon rank-sum Bonferroni’s adjusted *P* < 0.05) between clusters of interest was performed using the preranked workflow with gene set enrichment analysis (GSEA) software and the Molecular Signature Database (MSigDB) from the Broad Institute (46). Significantly differentially expressed genes were ranked by their average log-fold change in expression between groups. The gene expression data have been deposited in the NCBI’s Gene Expression Omnibus (GEO) database (GEO GSE154109).

### Statistics.

Statistical analysis of mass cytometry data was performed using 2-dimensional graphing and statistics software GraphPad Prism. Lack of Gaussian distribution was evaluated with the Shapiro-Wilk test. A nonparametric Mann-Whitney significance threshold of *P* < 0.05 was used to compare different cohorts. Wilcoxon’s rank-sum test with a significance threshold of *P* < 0.05 after Bonferroni’s correction was used to identify differentially expressed genes between clusters and disease states in the scRNA-Seq data. Data in graphs were plotted as mean ± SEM.

In order to identify risk cohorts, expression data across 34 parameters from mass cytometry panel within B-ALL (*n* = 36) and AML (*n* = 28) samples were used to identify patient clusters with similar expression profiles across parameters. Patient-to-patient similarities were assessed using Euclidean distances, and clusters were identified using Ward’s hierarchical agglomerative clustering method. Optimal number of clusters was determined using elbow (number of clusters that minimizes total intracluster variation) and silhouette methods. Within each sample, we further identified clusters with risk status (HR and SR) of patients. The association was ascertained by calculating risk ratio (RR) using Wald`s unconditional maximum likelihood estimation method. The significance of association was achieved at 0 < RR < 1 or RR > 1, and *P* < 0.05. Next, we used logistic regression model to identify markers significantly (0 < OR < 1 or OR > 1, and *P* < 0.05) associated with the clusters. The above analysis was performed using R version 3.6.1.

### Study approval.

Written institutional IRB-approved informed consent, if appropriate; patient assent; and consent from parent/legal guardian was obtained from all patients before obtaining samples used in the studies described (Yale/Emory), in accordance with the Declaration of Helsinki.

## Author contributions

JKB, SSM, KP, and JCV designed and performed experiments, analyzed data, and wrote the manuscript. DBD and AD performed experiments and analyzed data. AK, HSL, MLK, and PQ analyzed data and edited the manuscript. SC and DD collected patient material and edited the manuscript. CF performed experiments. MVD analyzed data, designed experiments, and edited the manuscript. KMD designed and supervised the study, analyzed data, and wrote the manuscript.

## Supplementary Material

Supplemental data

## Version 1 07/21/2020

In-Press Preview

## Version 2 08/20/2020

Electronic publication
