# Supplementary material for: Risk-associated alterations in marrow T cells in pediatric leukemia
Source: JCI Insight. 2020 Aug 20;5(16):e140179. doi: 10.1172/jci.insight.140179 (PMC7455136; doi:10.1172/jci.insight.140179)
Supplement: Supplemental data [file jciinsight-5-140179-s196.pdf]

Supplementary Data

Table of Contents

**Tables**	
Table S1	Summary of Patient Characteristics
Table S2	Mass Cytometry Antibodies
**Figures**	
Figure S1	Mass Cytometry Phenotypes of Bone Marrow T Cells in Childhood Leukemia.
Figure S2	SPADE Analysis of Bone Marrow T Cells
Figure S3	Immune Clusters Based on Bone Marrow T Cell Phenotypes in AML
Figure S4	Heatmap of Single-Cell RNA Sequencing Differential Gene Expression and T Cell Pathway Analysis

Table S1. Summary of patient characteristics

	B-ALL (n=36)	AML (n=28)	HD (n=11)
Age mean (range)	8 (2-18)	10.5 (1.6-18)	23 (18-30)
Gender			
Male	20 (56%)	16 (57%)	7 (64%)
Female	16 (44%)	12 (43%)	4 (36%)
Risk (B-ALL)[1]			
HR	14 (39%)	-	-
SR	22 (61%)	-	-
Risk (AML)[2]			
HR	-	14 (50%)	-
LR	-	14 (50%)	-
CNS Positive	8 (22%)	8 (29%)	-
Cytogenetics			
ETV6-RUNX1	8 (22%)	-	-
Trisomy (4, 10)	3 (8%)	-	-
inv(16)/t(16;16)		7 (25%)	
11q23 (KMT2A)	-	3 (11%)	
t(8;21)(q22;q22)	-	5 (18%)	
Monosomy 7	-	1(4%)	
Molecular Alterations			
FLT3-ITD	-	10 (36%)	-
NPM1 mutated		2(7%)	
MRD status End Induction I (n=28)			
CR	-	14 (50%)	-
PR	-	3 (10%)	-
PD	-	11 (40%)	-

Abbreviations: HD = healthy donor, HR = high-risk, SR = standard risk, LR = low-risk, MRD = minimal residual disease, CR = complete remission (<5% blasts), PR = partial remission (5-15% blasts), PD = persistent disease (>15% blasts).

Risk Classification[1,2]: B-ALL high-risk: Age $\geq$10 years; WBC$\geq$50,000/µl; B-ALL standard risk: Age 1.0-9.99 years; WBC $\leq$50,000/µl, based on findings at presentation. AML-low risk: favorable genetics including t(8:21); inv16, mutated NPM1 with wild-type FLT3, mutated CEBPA (normal karyotype). AML- high risk: Unfavorable genetics including FLT3-high internal tandem duplication allelic ratio, monosomy 7, monosomy 5, 5q deletions, KMT2A rearrangements, complex karyotype and residual disease following induction therapy.

Table S2. Mass Cytometry Antibodies.

	**Antibody**	**Clone**	**Supplier**		**Antibody**	**Clone**	**Supplier**
1	4-1BB	4B4-1	Biolegend	24	CD95	DX2	Fluidigm
2	BTLA	MIH26	Fluidigm	25	CTLA4	14D3	Fluidigm
3	CCR7	G043H7	Fluidigm	26	CXCR5	12G5	Fluidigm
4	CD103	Ber-ACT8	Fluidigm	27	DNAM1	TX25	Biolegend
5	CD11c	Bu15	Fluidigm	28	FOXP3	PCH101	Fluidigm
6	CD127	A019D5	Fluidigm	29	GATA3	TWAJ	Fluidigm
7	CD14	RM052	Fluidigm	30	Granzyme	GB11	Fluidigm
8	CD16	3G8	Fluidigm	31	HLA-DR	L243	Fluidigm
9	CD19	HIB19	Fluidigm	32	ICOS	C398.4A	Fluidigm
10	CD200	OX-104	Fluidigm	33	Ki67	Ki-67	Fluidigm
11	CD25	2A3	Fluidigm	34	LAG3	11C3C65	Fluidigm
12	CD27	L128	Fluidigm	35	NKG2A	Z199	Beckman Coulter
13	CD3	UCHT1	Fluidigm	36	NKG2C	134591	R&D Systems
14	CD33	WM53	Fluidigm	37	NKG2D	OW72	Fluidigm
15	CD38	HIT2	Fluidigm	38	OX40	ACT35	Fluidigm
16	CD4	RPA-T4	Fluidigm	39	PD1	EH12.2H7	Fluidigm
17	CD45	HI30	Fluidigm	40	PDL1	29E.2A3	Fluidigm
18	CD45RA	HI100	Fluidigm	41	Perforin	B-D48	Fluidigm
19	CD45RO	UCHL1	Fluidigm	42	T-Bet	4B10	Fluidigm
20	CD56	HCD56	Biolegend	43	TCF1	7F11A10	Biolegend
21	CD57	HCD57	Biolegend	44	TIGIT	MBSA43	Fluidigm
22	CD69	FN50	Fluidigm	45	TIM3	F38-2E2	Fluidigm
23	CD8	RPA-T8	Fluidigm				

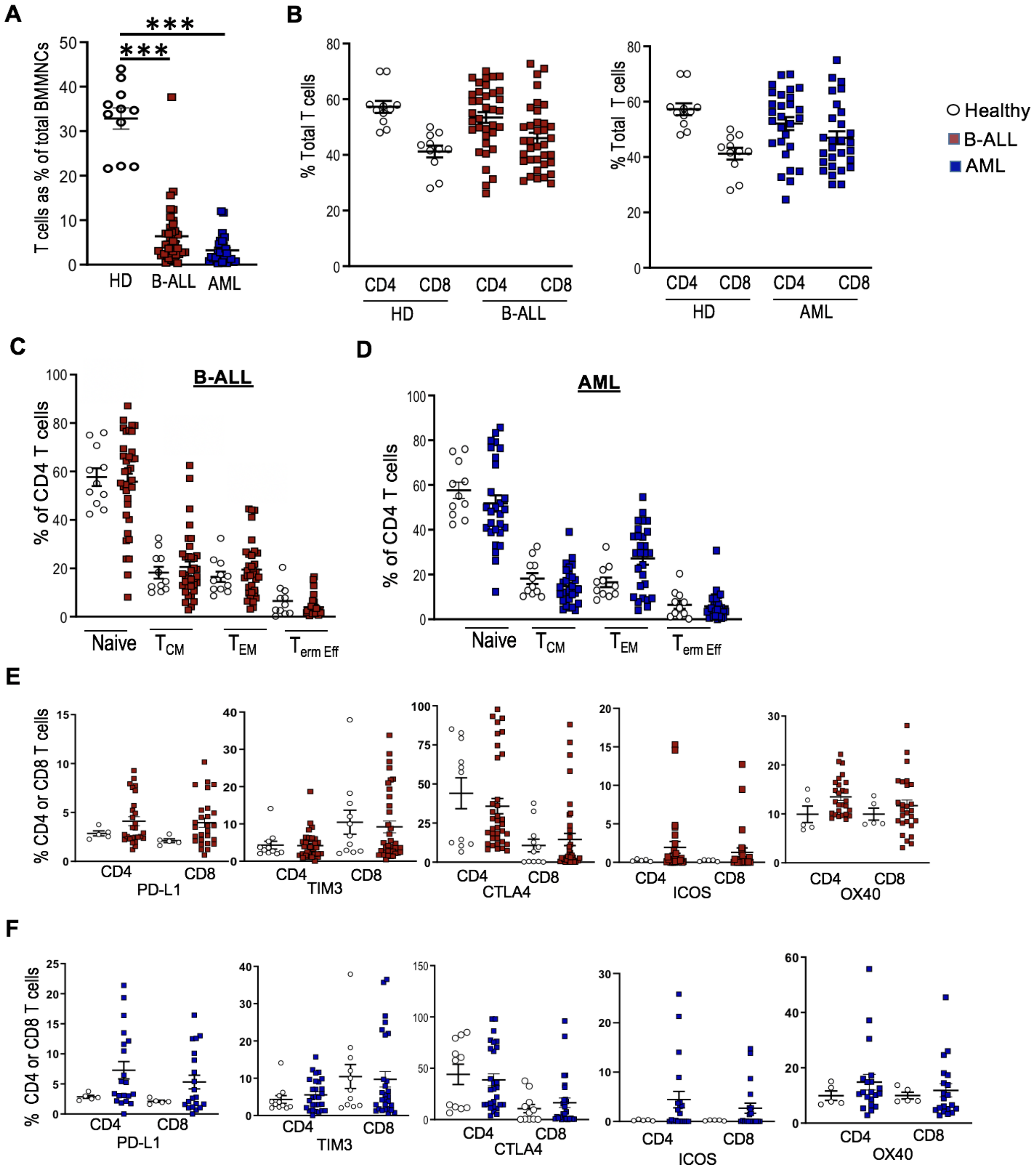
A
B
C
D
E
F
***
T cells as % of total BMMNCs
HD
B-ALL
AML
% Total T cells
CD4
CD8
Healthy
B-ALL
AML
% of CD4 T cells
Naive
T CM
T EM
T erm Eff
% CD4 or CD8 T cells
PD-L1
TIM3
CTLA4
ICOS
OX40

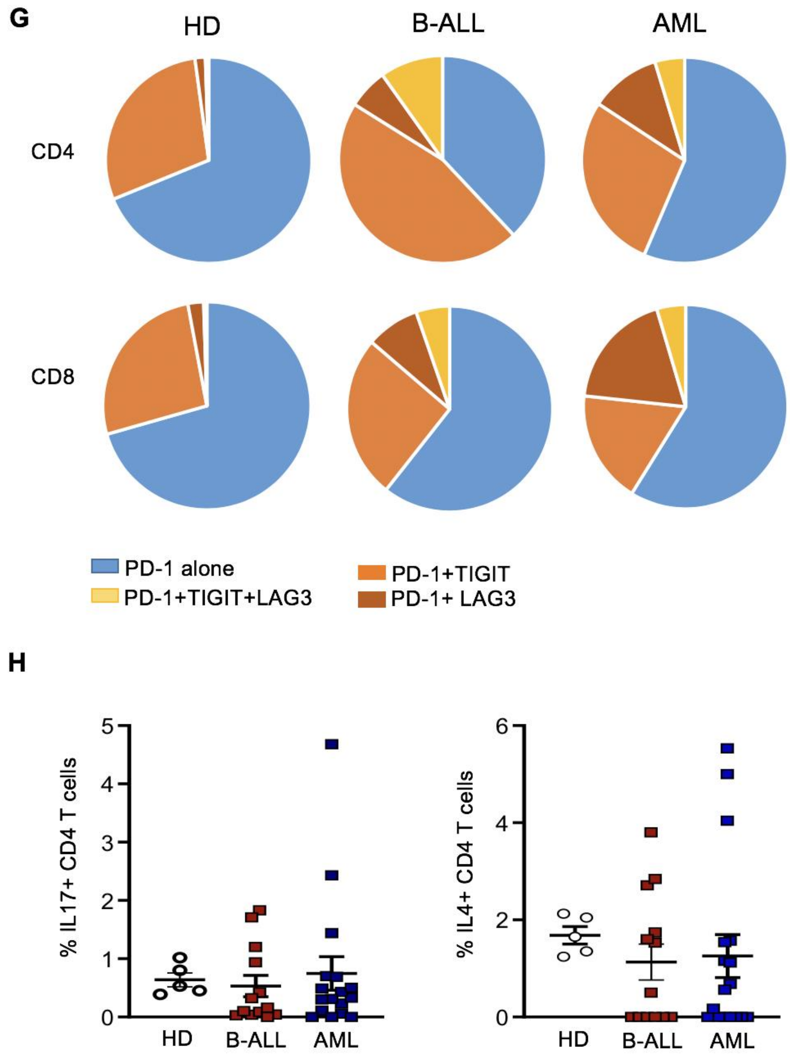

Figure S1. Mass Cytometry Phenotypes of Bone Marrow T Cells in Childhood Leukemia.

Bone marrow mononuclear cells (BMMNCs) from patients with B-ALL (n=36). AML (n=28) and healthy donors (HD) (n=5-11) were characterized using single cell mass cytometry. **(A)** T cells as percent of total BMMNCs in HD, B-ALL, and AML bone marrow. **(B)** CD4 and CD8 T cells as percent of total T cells in HD, B-ALL, and AML bone marrow. **(C)** Naïve, central memory (T_{CM}), effector memory (T_{EM}) and terminal effectors ($T_{ERM\ EFF}$) CD4 T cells as percent of total CD4 T cells in B-ALL bone marrow. **(D)** Naïve, central memory (T_{CM}), effector memory (T_{EM}) and terminal effectors ($T_{ERM\ EFF}$) CD4 T cells as percent of total CD4 T cells in AML bone marrow. **(E)** Percent of total CD4 or CD8 T cells expressing PD-L1, TIM3, CTLA4, ICOS, and OX40 in B-ALL and HD bone marrow (n=5 for PD-L1, ICOS, OX40). **(F)** Percent of total CD4 or CD8 T cells expressing PD-L1, TIM3, CTLA4, ICOS, and OX40 in AML and HD bone marrow (n=5 for PD-L1, ICOS, OX40). **(G)** Proportion of PD-1-expressing T cells co-expressing other inhibitory checkpoint molecules TIGIT and/or LAG3 in HD, B-ALL, and AML bone marrow. **(H)** Percent of total CD4 T cells expressing IL17 and IL4 in HD (n=5), B-ALL, and AML bone marrow. ***p<0.001, Mann-Whitney test.

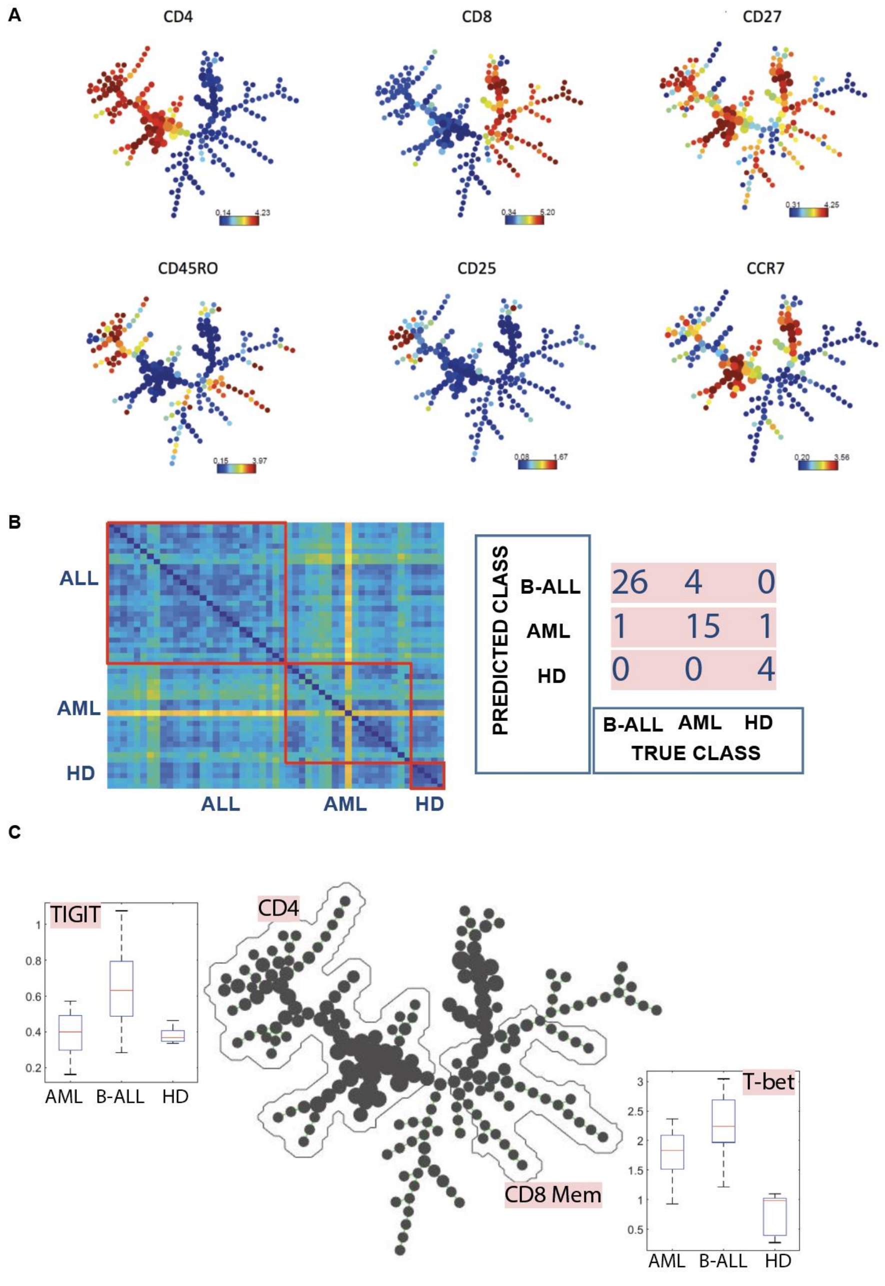

Figure S2. SPADE Analysis of Bone Marrow T Cells.

(A) Spanning tree progression analysis for density normalized events (SPADE) trees were generated showing expression of T cell surface markers measured by mass cytometry. These colored regions were used to partition the tree based on known T cell phenotypes. **(B)** Heatmap and confusion table showing ability of SPADE analysis to distinguish B-ALL, AML, and HD samples based on T cells alone. **(C)** SPADE tree showing different nodes with each node representing a unique T cell subset. Figure demonstrates TIGIT expression on CD4 T cells (B-ALL vs AML p=2.4*10-6, signal to noise ratio or SNR=1.4; and B-ALL vs HD p=0.004, SNR=1.4) and T-bet expression in CD8 memory T cells (B-ALL vs HD p=6.4*10-8, SNR=3.4; and AML vs HD p=8.3*10-5, SNR=2.3).

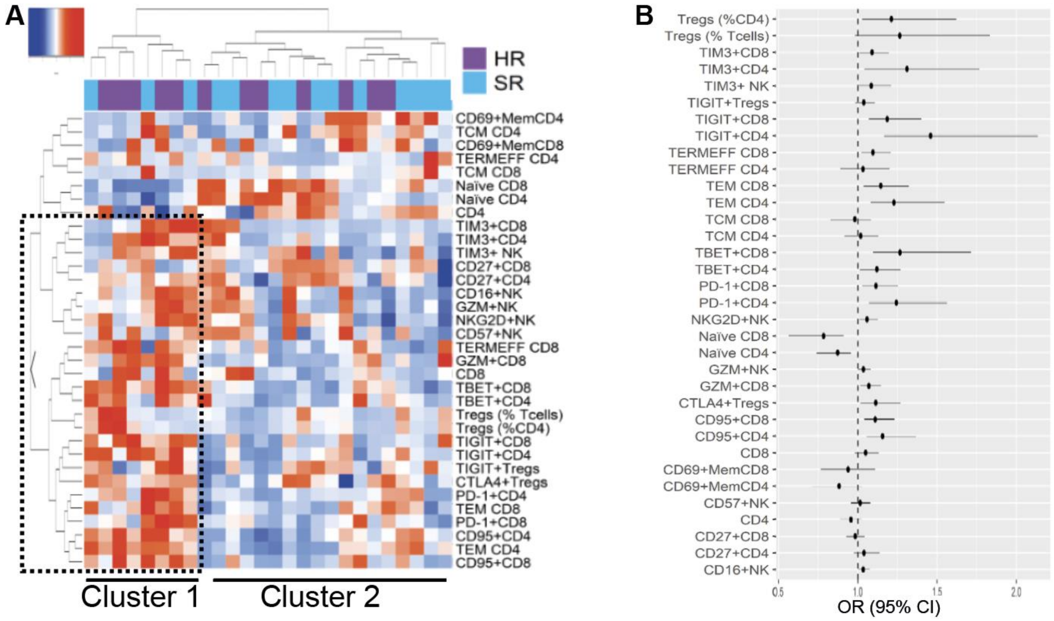

Figure S3. Immune Clusters Based on Bone Marrow T Cell Phenotypes in AML.

(A) Hierarchical cluster analysis based on mass cytometry immune markers in AML. **(B)** Forest plot showing regression analysis of markers linked to immune clusters in AML.

A

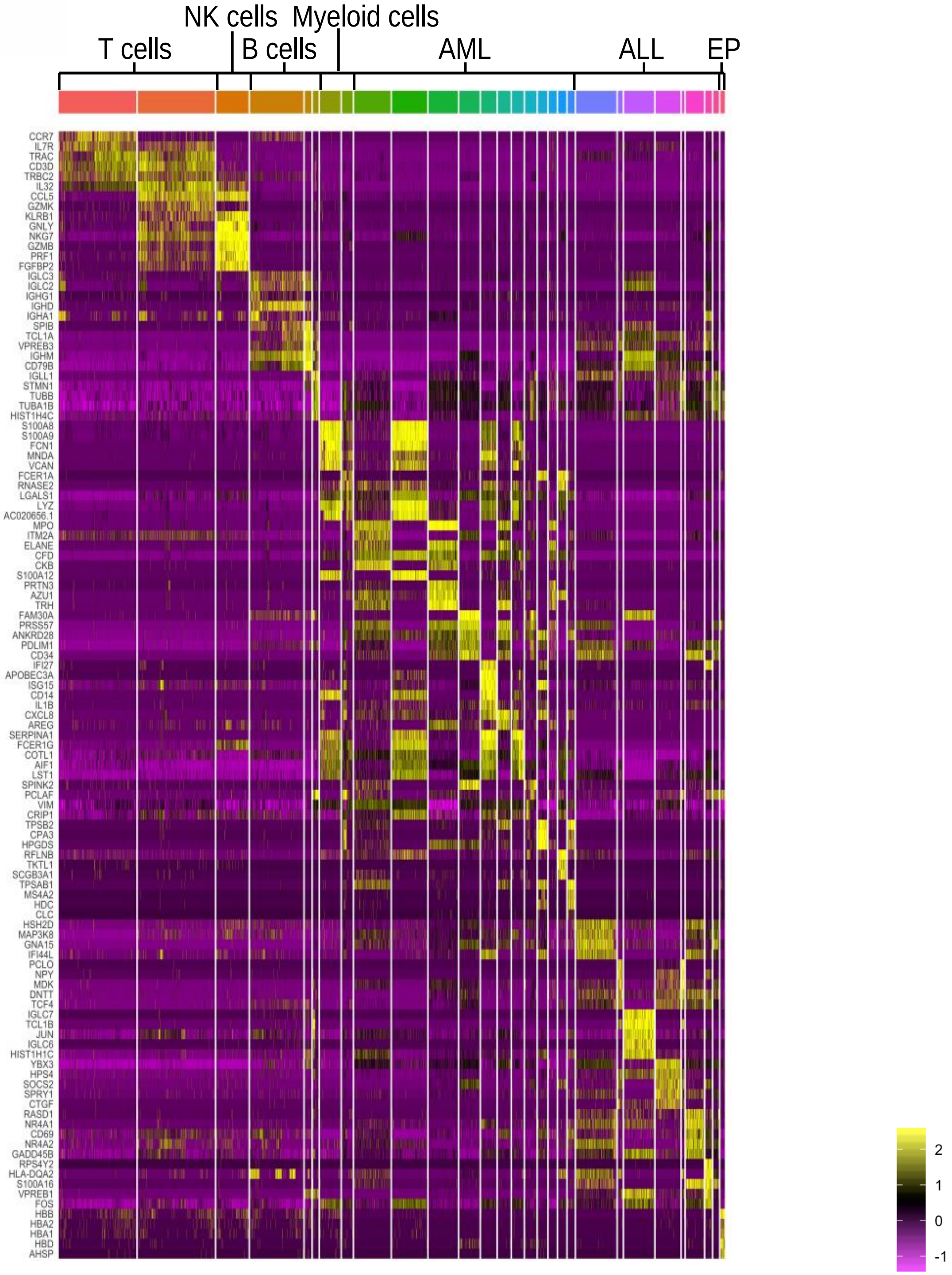
NK cells
Myeloid cells
T cells
B cells
AML
ALL
EP
CCR7
IL7R
TRAC
CD3D
TRBC2
IL32
CCL5
GZMK
KLRB1
GNLY
NKG7
GZMB
PRF1
FGFBP2
IGLC3
IGLC2
IGHG1
IGHD
IGHA1
SPIB
TCL1A
VPREB3
IGHM
CD79B
IGLL1
STMN1
TUBB
TUBA1B
HIST1H4C
S100A8
S100A9
FCN1
MNDA
VCAN
FCER1A
RNASE2
LGALS1
LYZ
AC020656.1
MPO
ITM2A
ELANE
CFD
CKB
S100A12
PRTN3
AZU1
TRH
FAM30A
PRSS57
ANKRD28
PDLIM1
CD34
IFI27
APOBEC3A
ISG15
CD14
IL1B
CXCL8
AREG
SERPINA1
FCER1G
COTL1
AIF1
LST1
SPINK2
PCLAF
VIM
CRIP1
TPSB2
CPA3
HPGDS
RFLNB
TKTL1
SCGB3A1
TPSAB1
MS4A2
HDC
CLC
HSH2D
MAP3K8
GNA15
IFI44L
PCLO
NPY
MDK
DNTT
TCF4
IGLC7
TCL1B
JUN
IGLC6
HIST1H1C
YBX3
HPS4
SOCS2
SPRY1
CTGF
RASD1
NR4A1
CD69
NR4A2
GADD45B
RPS4Y2
HLA-DQA2
S100A16
VPREB1
FOS
HBB
HBA2
HBA1
HBD
AHSP
2
1
0
-1

B

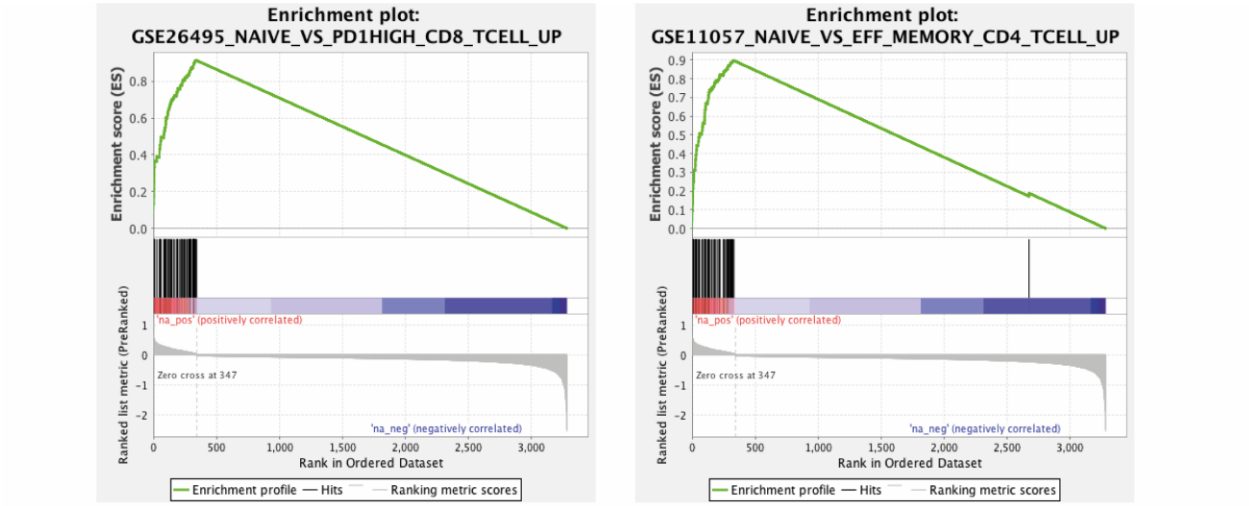

Gene set ID	Enriched Cluster	Description	Nominal p-value	PMID
GSE26945 (shown above left)	T1	Genes up-regulated in naïve vs PD-1 high CD8 T cells	p<0.0001	21383243
GSE11057 (shown above right)	T1	Genes up-regulated in naïve vs effector memory CD4 T cells	p<0.0001	19568420
KAECH	T2	Genes up-regulated in effector vs naïve CD8 T cells	p<0.0001	12526810
GSE45739	T2	Genes upregulated in activated vs unstimulated CD4 T cells	p<0.0001	23755101

Figure S4. Heatmap of Single-Cell RNA Sequencing Differential Gene Expression and T Cell Pathway Analysis.

(A) Zero-centered gene expression of significantly (Wilcoxon rank-sum test with Bonferroni correction p<0.05) highly differentially expressed genes defining clusters (selected by largest average log-fold change in expression between each cluster and all other cells). NK=natural killer, AML=acute myeloid leukemia, ALL=acute lymphoblastic leukemia, EP=erythroid progenitor. **(B)** GSEA pathway analysis of significantly differentially expressed genes (Wilcoxon rank-sum test p<0.05 with Bonferroni correction) between T cell clusters T1 (enriched in HD and low-risk ALL) and T2 (enriched in high-risk ALL and AML) revealed up-regulation of pathways associated with naïve T cells in cluster T1 relative to pathways associated with PD-1 high and effector T cells in cluster T2.
